# Small-Molecule Inhibition of BRDT for Male Contraception

**DOI:** 10.1016/j.cell.2012.06.045

**Published:** 2012-08-17

**Authors:** Martin M. Matzuk, Michael R. McKeown, Panagis Filippakopoulos, Qinglei Li, Lang Ma, Julio E. Agno, Madeleine E. Lemieux, Sarah Picaud, Richard N. Yu, Jun Qi, Stefan Knapp, James E. Bradner

**Affiliations:** 1 Department of Pathology & Immunology, Baylor College of Medicine, Houston, TX 77030, USA; 2 Department of Molecular and Cellular Biology, Baylor College of Medicine, Houston, TX 77030, USA; 3 Department of Molecular and Human Genetics, Baylor College of Medicine, Houston, TX 77030, USA; 4 Department of Pharmacology, Baylor College of Medicine, Houston, TX 77030, USA; 5 Center for Drug Discovery, Baylor College of Medicine, Houston, TX 77030, USA; 6 Center for Reproductive Medicine, Baylor College of Medicine, Houston, TX 77030, USA; 7 Department of Medical Oncology, Dana-Farber Cancer Institute, Boston, MA 02215, USA; 8 Nuffield Department of Clinical Medicine, Structural Genomics Consortium, University of Oxford, Oxford OX3 7DQ, UK; 9 Department of Veterinary Integrative Biosciences, College of Veterinary Medicine & Biomedical Sciences, Texas A&M University, College Station, TX 77843, USA; 10 Bioinfo, Ottawa, Ontario K1J 8G4, Canada; 11 Department of Urology, Children’s Hospital Boston, Boston, MA 02115, USA; 12 Department of Biochemistry and Molecular Biology, George Washington University, School of Medicine and Health Sciences, Washington, DC 20037, USA; 13 Department of Medicine, Harvard Medical School, Boston, MA 02115, USA

## Abstract

A pharmacologic approach to male contraception remains a longstanding challenge in medicine. Toward this objective, we explored the spermatogenic effects of a selective small-molecule inhibitor (JQ1) of the bromodomain and extraterminal (BET) subfamily of epigenetic reader proteins. Here, we report potent inhibition of the testis-specific member BRDT, which is essential for chromatin remodeling during spermatogenesis. Biochemical and crystallographic studies confirm that occupancy of the BRDT acetyl-lysine binding pocket by JQ1 prevents recognition of acetylated histone H4. Treatment of mice with JQ1 reduced seminiferous tubule area, testis size, and spermatozoa number and motility without affecting hormone levels. Although JQ1-treated males mate normally, inhibitory effects of JQ1 evident at the spermatocyte and round spermatid stages cause a complete and reversible contraceptive effect. These data establish a new contraceptive that can cross the blood:testis boundary and inhibit bromodomain activity during spermatogenesis, providing a lead compound targeting the male germ cell for contraception.

**PaperClip:**

## Graphical abstract

**Graphical Abstract:**

**Figure:**
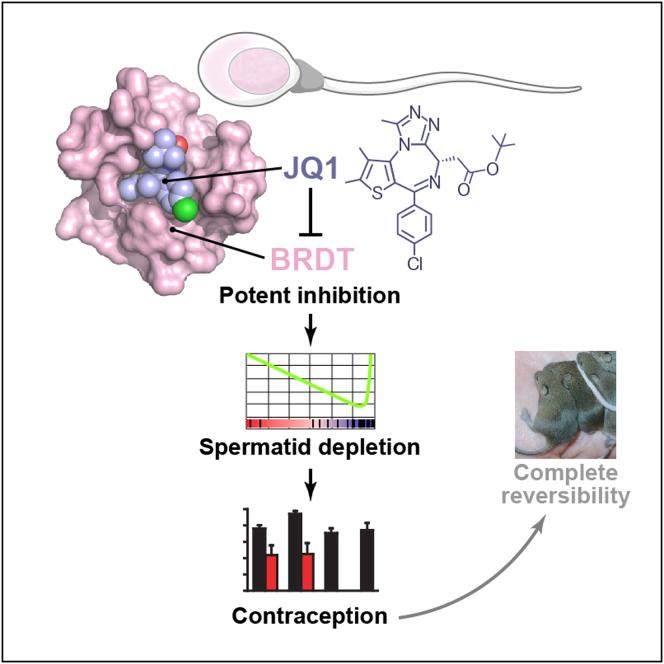

**Highlights:**

► Bromodomain, testis-specific (BRDT) is a contraceptive target ► JQ1 is a BRDT inhibitor that causes a reversible contraceptive effect in male mice ► JQ1 alters spermatogenesis at the spermatocyte and round spermatid stages ► JQ1 treatment targets the male germline and reduces spermatozoa number and motility

## Teaser

Inhibition of the chromatin reader protein BRDT with the small molecule JQ1 provides an approach for reversible male contraception.

## Introduction

Although ∼4% of the mammalian genome encodes genes expressed in male germ cells during spermatogenesis (Schultz et al., 2003), contraceptive drugs for men have remained elusive. To date, the only drugs in clinical trials are testosterone analogs that alter endogenous androgen production, although there is a short list of other possible targets (e.g., GAPDHS) and drugs (e.g., gamendazole) (Aitken et al., 2008). This lack of contraceptive alternatives for men is partially responsible for the high rate of unplanned pregnancies, especially in teenagers, and contributes to the maternal mortality, ethical, social, and financial costs associated with abortions and deliveries to single mothers. To approach this dearth of contraceptive alternatives for men, we have undertaken to develop small molecules that could target spermatogenic-specific proteins that have been shown to be essential for both spermatogenesis and fertility in mammals. One such contraceptive target is the testis-specific and bromodomain-containing protein BRDT.

**Figure 1:**
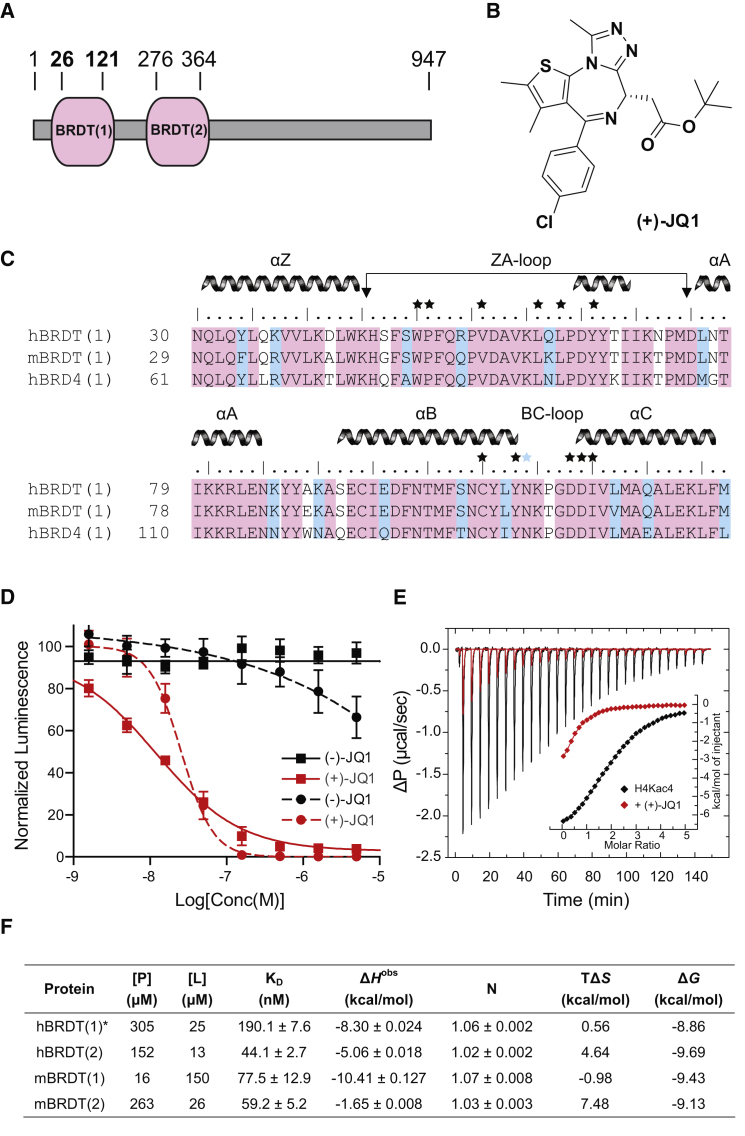
BRDT Inhibition by the BET Bromodomain Inhibitor JQ1 (A) Domain diagram of BRDT. Sequence boundaries for recombinant BRDT(1) are shown in bold. (B) Structure of the active (+)-JQ1 enantiomer. (C) Protein alignment reveals high sequence identity between homologous and orthologous domains. Identical (pink) and similar (blue) residues are highlighted. Major helical elements are depicted above the sequence. The conserved asparagine mediating acetyl-lysine recognition is depicted with a blue star. Contacts between (+)-JQ1 and BRDT(1) are depicted with a black star. (D) Competitive inhibition of human (squares) and mouse (circles) BRDT(1) binding to synthetic biotinylated H4Kac4 by (+)-JQ1 using proximity detection assays (hBRDT(1) IC_50_ = 11 nM; mBrdt(1) IC_50_ = 10 nM). (E) ITC data for titration of H4Kac4 into hBRDT(1) (black line) or into a 1:0.8 molar mixture of hBRDT(1) and (+)-JQ1 (red line). The inset shows normalized binding enthalpies corrected for heat of dilution as a function of binding site saturation. Solid lines represent a nonlinear least-squares fit using a single-site binding model. (F) Equilibrium binding constants and binding energies of (+)-JQ1 to human and mouse BRDT bromodomains measured by ITC. See also Data S1 and S2 and Table S1.

BRDT is a tissue-restricted, chromatin-associated protein expressed in pachytene spermatocytes, diplotene spermatocytes, and round spermatids (Shang et al., 2007). During postmeiotic maturation, BRDT localizes to the nucleus and reorganizes hyperacetylated histones through twin acetyl-lysine recognition modules, or bromodomains (Berkovits and Wolgemuth, 2011; Morinière et al., 2009; Shang et al., 2007). The essential role of BRDT in spermatogenesis is mediated by the first bromodomain (BRDT(1); Figure 1A), which binds the tetra-acetylated amino-terminal tail of histone 4 (H4Kac4) with moderate potency (20 μM) (Morinière et al., 2009). Structural studies of murine BRDT have demonstrated that BRDT(1) binds a diacetylated histone 4 peptide (H4K5ac8ac) in part through a conserved asparagine (Morinière et al., 2009), akin to other bromodomain coactivator proteins (Dhalluin et al., 1999). Genetic studies of BRDT have demonstrated that selective deletion of the BRDT(1)-encoding region is sufficient to confer sterility in homozygous hypomorphic male mice (Shang et al., 2007), and a recently published genome-wide association study of idiopathic male infertility identified single-nucleotide polymorphisms of *BRDT* as significantly associated with oligozoospermia or azoospermia in European men (Aston et al., 2010). These insights establish a compelling rationale to target BRDT for a contraceptive effect.

Recently, we established the feasibility of targeting human bromodomains with acetyl-lysine competitive small molecules (Filippakopoulos et al., 2010). Our index study described a potent thienodiazepine inhibitor ((+)-JQ1; Figure 1B; *K*_*d*_ = 90 nM) of the BET family coactivator protein BRD4, which is implicated in the pathogenesis of cancer via transcriptional control of the *MYC* oncogene (Delmore et al., 2011; Zuber et al., 2011). Protein sequence alignment of human BRD4(1) to human BRDT(1) reveals 81% identity and 89% similarity, including all surface residues predicted to contact (+)-JQ1 (Figure 1C and Data S1 and S2 available online). Based on these insights and preliminary evidence of binding to BRDT(1) established by differential scanning fluorometry (Filippakopoulos et al., 2010), we endeavored to characterize the biochemical and functional effects of (+)-JQ1 on BRDT(1).

We report here that direct inhibition of BRDT by a small-molecule bromodomain inhibitor exerts a dose- and time-dependent inhibitory effect on spermatogenesis. Structural studies of BRDT(1) bound to JQ1 reveal ligand-receptor shape complementarity and complete occlusion of the acetyl-lysine recognition cavity. These and further biochemical studies establish the molecular mechanism of potent BRDT inhibition observed in vivo, where JQ1 targets meiotic and postmeiotic male germ cells, leading to impaired spermatogenesis and compromised motility. In mating studies, JQ1 accomplishes a complete and reversible contraceptive effect in males without adversely affecting testosterone levels or mating behaviors and without prompting obvious teratogenic effects in offspring. These results indicate that targeting a developmental epigenetic reader protein with an orally bioavailable small molecule can modulate male fertility for a contraceptive effect.

## Results

### Molecular Recognition of BRDT by JQ1

To assess competitive binding to BRDT(1), we devised homogeneous, luminescence proximity assays that are capable of quantifying binding of a synthetic, biotinylated tetra-acetylated histone 4 peptide (H4Kac4, residues 1–20) to recombinant epitope-tagged murine or human BRDT(1). Dose-ranging studies of (+)-JQ1 demonstrated potent inhibition of H4Kac4 binding with a half-maximum inhibitory concentration (IC_50_) value of 10 nM for murine BRDT(1) and 11 nM for human BRDT(1) (Figure 1D). In contrast, the (−)-JQ1 enantiomer was inactive for either ortholog, establishing stereospecific, ligand-competitive binding. To confirm competitive inhibition of the acetyl-lysine binding module, isothermal titration calorimetry (ITC) was performed on human BRDT(1), employing a synthetic H4Kac4 peptide in the presence and absence of (+)-JQ1 (Figure 1E). This peptide was found to bind two protein modules concomitantly with a *K*_*d*_ of 25.5 μM, in good agreement with published data (Morinière et al., 2009). This interaction was directly inhibited in the presence of (+)-JQ1 that partially saturated the acetyl-lysine binding (ratio 1:0.8) due to solubility limits of the inhibitor. The remaining unoccupied sites bound the H4Kac4 peptide with identical affinity and expected stoichiometry (Table S1). Binding free energies and dissociation constants (K_D_) determined by ITC for each bromodomain of human and murine BRDT confirm potent, specific (ratio 1:1) interactions with all domains (K_D_ = 44–190 nM; Figure 1F). Notably, (+)-JQ1 binds murine BRDT(1) with high ligand efficiency (LE = 0.30), compared to reported inhibitors of protein-protein interactions (Wells and McClendon, 2007).

**Figure 2:**
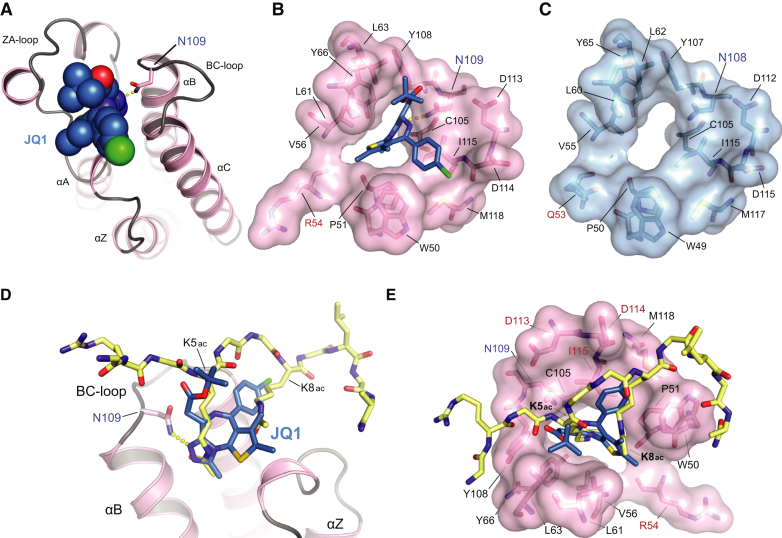
Molecular Recognition of BRDT by JQ1 (A) Crystal structure of the complex of (+)-JQ1 with hBRDT(1). The ligand is shown as a Corey-Pauling-Koltun (CPK) model, and the hydrogen bond formed to the conserved asparagine (N109) is shown as yellow dots. Main secondary structural elements are labeled. (B) The acetyl-lysine binding pocket of the N-terminal bromodomain of hBRDT is shown as a semitransparent surface with contact residues labeled and depicted in stick representation. Carbon atoms of (+)-JQ1 are colored blue to distinguish them from protein residues. N109 is labeled in blue, and a unique N-terminal residue is shown in red. (C) The surface structure of the acetyl-lysine recognition pocket of mBrdt(1) is shown in blue, with key residues highlighted in black, N109 highlighted in blue, and a distinct residue shown in red. (D and E) Competitive binding of JQ1 to the acetyl-lysine recognition pocket of hBRDT(1) illustrated by structural alignment to an acetylated histone peptide binding to mBrdt(1) (PDB: 2WP2). (D) Human BRDT(1) is shown as pink ribbons. JQ1 is shown as blue sticks with colored heteroatoms. N109 is highlighted in blue. The diacetylated histone H4 peptide is shown in yellow with colored heteroatoms. (E) The diacetyl-lysine recognition site on hBRDT is shown as a pink translucent surface over stick representations of critical binding residues. Key contact residues are highlighted in black. JQ1 is shown as blue sticks with colored heteroatoms. N109 is highlighted in blue text. The diacetylated histone H4 peptide is shown in yellow with colored heteroatoms. (D and E) JQ1 completely occupies the hydrophobic pocket which engages the diacetylated peptide. See also Table S2.

The mode of binding of (+)-JQ1 to BRDT(1) was established by X-ray crystallography at high-resolution (Figure 2 and Table S2). Similar to the structure of murine BRDT(1) (Morinière et al., 2009), human BRDT(1) exhibits the canonical structural features of a bromodomain, namely a left-handed bundle of four antiparallel α helices (αZ, αA, αB, and αC) and a hydrophobic acetyl-lysine binding pocket defined by interhelical ZA and BC loops (Figures 2A–2C) (Dhalluin et al., 1999). The observed, extraordinary shape complementarity between (+)-JQ1 and BRDT(1) explains the increased potency of (+)-JQ1 compared to the physiologic H4Kac4 ligand. In addition, (+)-JQ1 engages all surface residues analogous to those on murine BRDT(1) mediating molecular recognition of H4K5ac8ac (Figures 2D and 2E) (Morinière et al., 2009). Notable examples include a hydrogen bond linking the triazole ring and the evolutionarily conserved asparagine (Asn109), as well as extensive hydrophobic contacts with the ZA and BC loop regions (Figure 2B). These biochemical and structural studies confirm and explain potent, acetyl-lysine competitive inhibition of BRDT(1) by (+)-JQ1 (hereafter referred to as JQ1).

### Testis Bioavailability of JQ1

**Figure 3:**
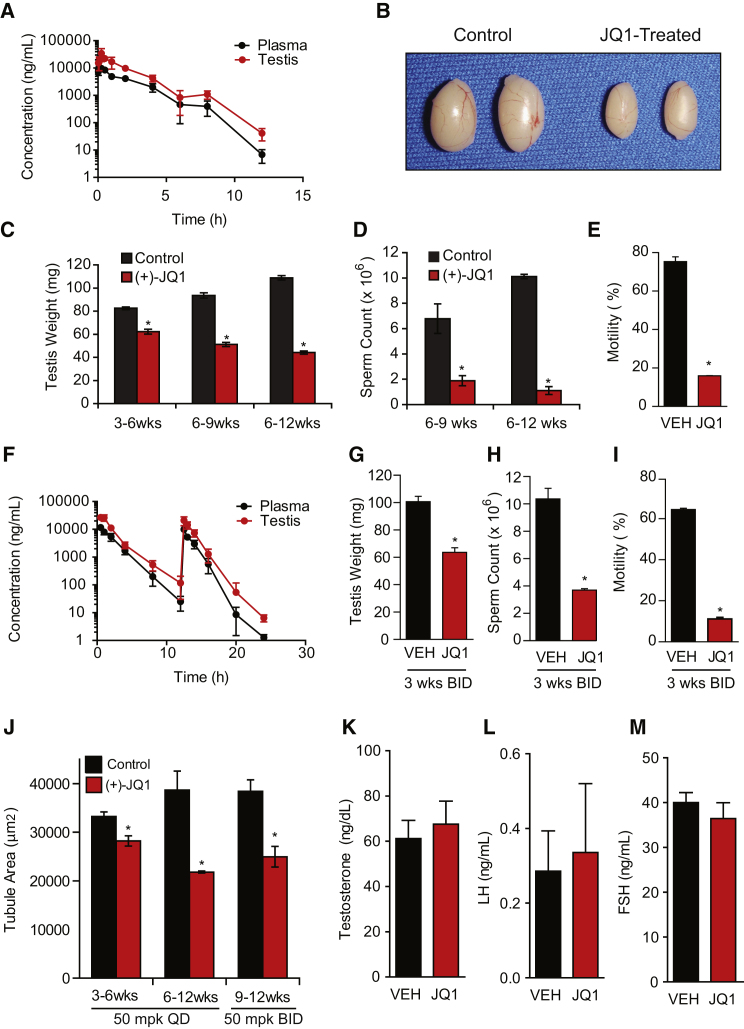
BRDT Inhibition with a Testis-Permeable Small Molecule Reduces Testis Size and Sperm Counts and Motility (A) Pharmacokinetic analysis of JQ1 in plasma (black lines) and testis (red lines) following a single administration of JQ1 (50 mg/kg IP) to male mice. Data represent the mean ± SD. (B) Gross analysis of testes from 9-week-old mice that received control or JQ1 (50 mg/kg daily) for 3 weeks. (C) Testis weights from mice treated with control or JQ1 (50 mg/kg QD) for 3–6 weeks, 6–9 weeks, or 6–12 weeks of age. Data represent the mean ± SEM and are annotated with P values obtained from a two-tailed t test (^∗^p < 0.05). (D) Graphical representation of sperm counts obtained from the entire epididymides of males treated with JQ1 or control from 6–9 weeks of age or the tail of the epididymis of males treated from 6–12 weeks of age with vehicle or JQ1 (50 mg/kg QD). Data represent the mean ± SEM (^∗^p < 0.05). (E) Motility of mature sperm obtained from the cauda epididymis of adult males treated with JQ1 (50 mg/kg daily) from 6–12 weeks of age. Data represent the mean ± SEM (^∗^p < 0.0001). (F) Pharmacokinetic analysis of JQ1 in plasma (black lines) and testis (red lines) following twice-daily (BID) administration of JQ1 (50 mg/kg IP) to male mice. Data represent the mean ± SD. (G) Testis weights (mg) from mice treated with control or JQ1 (50 mg/kg BID) from 9–12 weeks of age. Data represent the mean ± SEM (^∗^p < 0.05). (H) Sperm counts obtained from the cauda epididymis of males treated from 9–12 weeks of age with vehicle or JQ1 (50 mg/kg BID). Data represent the mean ± SEM (^∗^p < 0.05). (I) Motility of mature sperm obtained from the cauda epididymis of adult males treated with JQ1 (50 mg/kg BID) or vehicle from 9–12 weeks. Data represent the mean ± SEM (^∗^p < 0.001). (J) Cross-sectional area of seminiferous tubules from JQ1-treated mice (50 mg/kg QD for 3–6 weeks or 6–12 weeks, or 50 mg/kg BID for 9–12 weeks, as shown) or control (^∗^p < 0.05). (K, L, and M) Male mice treated with JQ1 or vehicle from 6–12 weeks exhibit statistically similar serum levels of (K) testosterone, (L) luteinizing hormone (LH), and (M) follicle-stimulating hormone (FSH). See also Figure S1 and Tables S3 and S4.

To test the ability of JQ1 to reach its contraceptive target cells, the testicular bioavailability of JQ1 was assessed by serum and tissue pharmacokinetic analysis. Following a single intraperitoneal (i.p.) dose of JQ1 (50 mg/kg) in male mice, measurements of JQ1 concentration were made in serum, testis, and brain (Figure 3A and Table S3). Overall, JQ1 exhibits excellent testicular bioavailability (AUC_testis_/AUC_plasma_ = 259%), suggesting preferential distribution into this tissue compartment with rapid (T_max_ = 0.25 hr) and pronounced exposure (C_max_ = 34 μg/ml). Corroborating barrier permeability by JQ1, nearly uniform blood-brain barrier permeability was observed after single-dose pharmacokinetic studies (AUC_brain_/AUC_plasma_ = 98%).

### JQ1 Impairs Sperm Count and Motility

To determine the possible consequences of blocking BRDT function in vivo, we tested the spermatogenic effects of JQ1 administered to male mice. First, juvenile or adult male mice were administered daily i.p. injections of JQ1 (50 mg/kg once daily [QD]) or vehicle control over a 3 or 6 week period. After drug or vehicle treatment, mice were either sacrificed or mated to females while continuing to receive JQ1. All JQ1-treated males had a significant reduction in testis volume (Figures 3B and 3C). With daily injections of 50 mg/kg JQ1, males treated from 3–6 weeks of age showed a reduction to 75.4% of control, males treated from 6–9 weeks of age showed a reduction to 54.7%, and males treated for 6 weeks with JQ1 (6–12 weeks of age) showed a reduction to 40.6% (Figure 3C). Thus, longer treatment with JQ1 has the most prominent effect on testicular volume.

**Figure S1:**
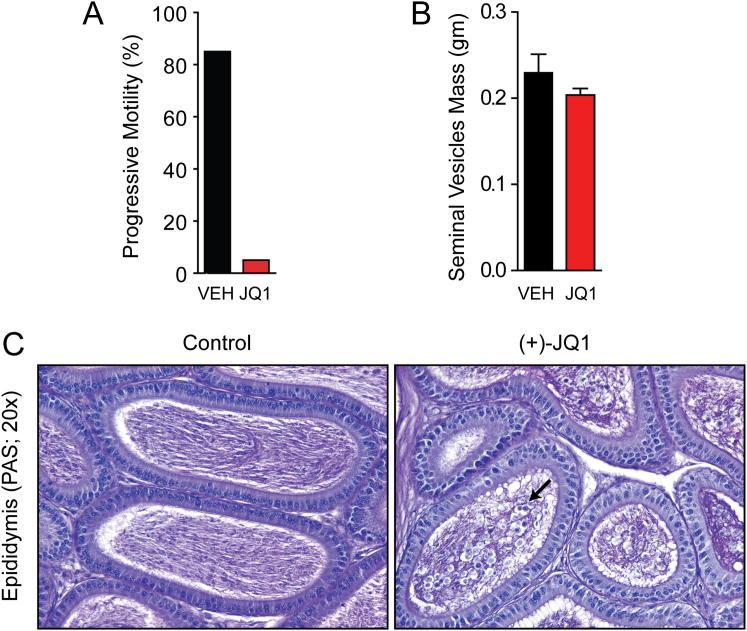
Motility and Histological Effect of JQ1 Treatment on the Epididymides of Male Mice, Related to Figure 3 (A) Progressive motility of mature sperm obtained from the epididymis of males treated with JQ1 (50 mg/kg QD) or control from 6-12 weeks of age. Data was obtained using video analysis of sperm on a 37°C heated stage. (B) Treatment of male mice with JQ1 (50 mg/kg QD) for 8 weeks does not affect the mass of seminal vesicles. (C) Histology of the epididymides from male mice treated with control or JQ1 (50 mg/kg QD) from 6-12 weeks of age. Fewer spermatozoa and multiple large nucleated cells (black arrow) are observed in the epididymal lumen of the JQ1-treated mice compared to the control epididymal lumen, which is densely packed with mature spermatozoa.

To examine the effect on sperm production, sperm counts were determined after JQ1 or vehicle treatment. Epididymal sperm number was reduced to 28% of control after 3 weeks of 50 mg/kg daily exposure (3–6 weeks of age) and 11% of control after 6 weeks of treatment (6–12 weeks of age) (Figure 3D). Analysis of sperm motility after 3 weeks of JQ1 (50 mg/kg QD) revealed a significant, 4.5-fold reduction in sperm motility (16.0% for the JQ1-treated versus 72.4% for the control; Figure 3E). Following 6 weeks of daily JQ1, only 5% of the sperm demonstrated progressive motility compared to 85% of sperm from controls (Figure S1). Thus, daily exposure to JQ1 quantitatively reduced sperm number and motility.

Recently, the tolerability of twice-daily administration of JQ1 was reported in a confirmatory study of JQ1 in hematologic malignancies (Mertz et al., 2011). Pharmacokinetic study of twice-daily (BID) JQ1 (50 mg/kg) confirmed excellent penetration of the blood:testis barrier and demonstrated a marked increase in drug exposure in the testis (AUC_0–24hr_ = 96,640 hr^∗^ng/ml; Figure 3F and Table S4), accompanied by apparent partitioning into this compartment (AUC_testis_/AUC_plasma_ = 236%). With BID injections of JQ1 (JQ1 50 mg/kg), adult males treated for 3 weeks (9–12 weeks) showed reduction in testis weight to 63.3% of controls (Figure 3G). Paralleling the reduction in testis weights, BID treatment resulted in reduction in sperm in the cauda epididymis to 35.6% of the control (Figure 3H), a dramatic 5.8-fold reduction in sperm motility (11.2% for JQ1-treated versus 64.5% for controls; Figure 3I), firmly establishing the tolerability and antispermatogenic effect of BID JQ1 in mice. Altogether, these findings are similar to defects observed in mice deficient in BRDT(1) (Shang et al., 2007).

### JQ1 Selectively Targets Male Germ Cells without Affecting Hormone Levels

**Figure 4:**
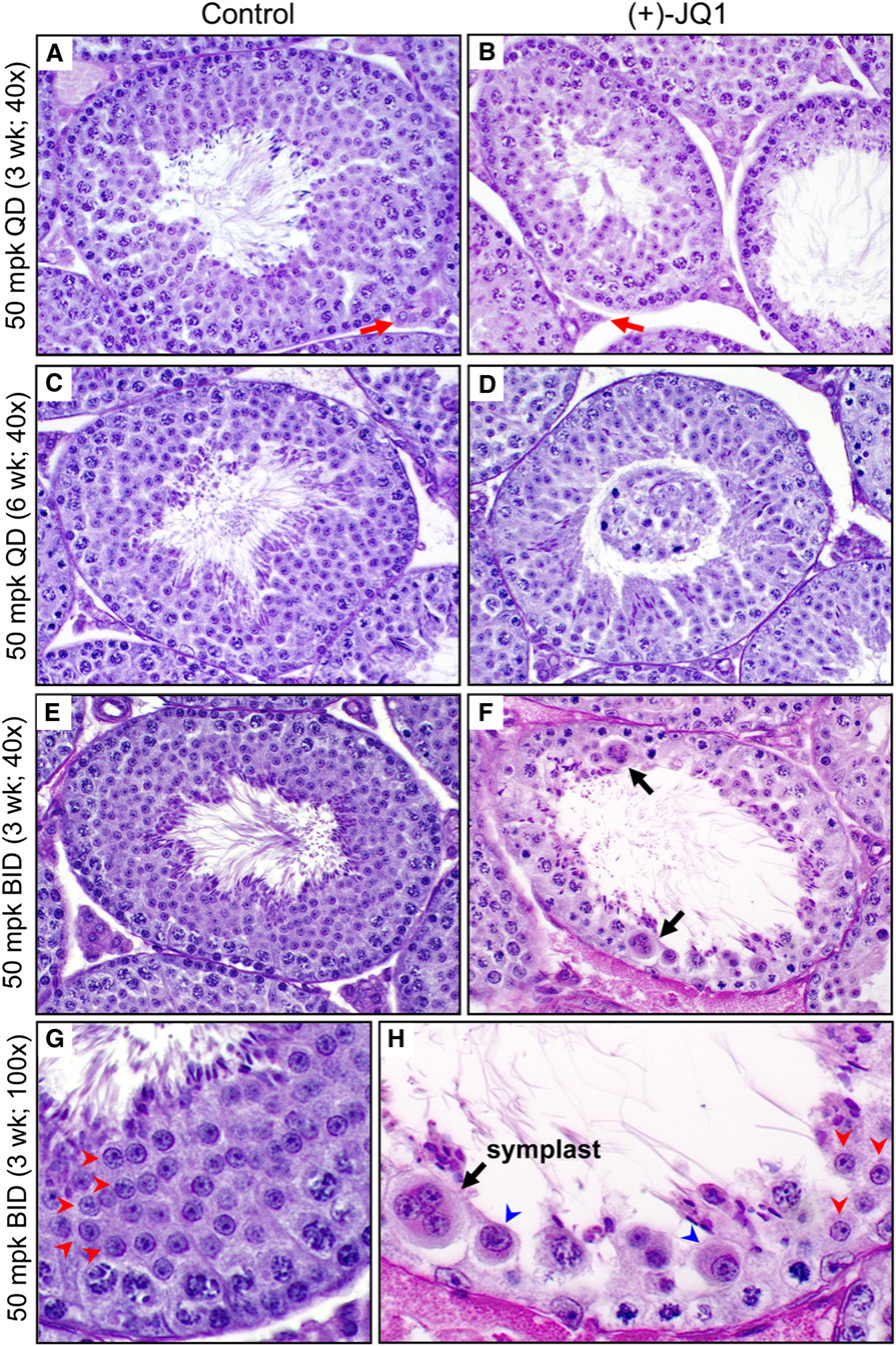
Histological Analysis of the Antispermatogenic Effects of JQ1 (A and B) Histology of stage VII seminiferous tubules of testes of 6-week-old mice treated with (A) vehicle control or (B) JQ1 (50 mg/kg QD) from 3–6 weeks of age (40× magnification). Red arrows indicate intertubular Leydig cell islands. (C and D) Histology of stage VII seminiferous tubules of 12-week-old mice treated with (C) control or (D) JQ1 (50 mg/kg QD) from 6–12 weeks. A large mass of sloughed epithelium is observed in the lumen of the tubule in (D). (E–H) Histological analysis of testis tubules from 12-week-old mice treated with vehicle control (E, 40×; G, 100×) or JQ1 (50 mg/kg BID) (F, 40×; H, 100×) for 3 weeks. Whereas the stage VII tubules of the control show an abundance of round spermatids (red arrowheads, G), only a few normal appearing round spermatids are evident in (H) after JQ1 treatment. Abnormal spermatids with large nuclei and abundant cytoplasm (blue arrowheads) and symplasts (black arrows in F and H) are also observed.

Based on the observed inhibition of spermatogenesis, we performed further mechanistic studies of BRDT inhibition. Consistent with the reduction in testis volume, the cross-sectional area of seminiferous tubules from testes of males treated daily from 3–6 weeks with JQ1 were 84.9% of controls, males treated from 6–12 weeks with JQ1 were 56.4%, and males treated from 9–12 weeks with JQ1 BID were 65.0% of controls (Figure 3J). Histological examination established a JQ1-dependent decrease in the amount and number of tubules that had obvious and abundant round spermatids and spermatozoa in the lumen (Figures 4A–4H). This was particularly apparent in stage VII tubules, in which a marked reduction in round spermatids was observed in JQ1-treated mice. Of note, mice treated with JQ1 BID for 3 weeks had a 79% reduction in round spermatids in stage VII tubules (112.7 ± 7.4 [controls] versus 23.9 ± 3.6 [JQ1-treated]; p < 0.0001). Analysis of epididymides of JQ1-treated males also showed a similar finding in which fewer sperm were observed in the epididymal lumen compared to their abundance in controls (Figure S1). Detailed microscopic analysis of the testes of mice treated with JQ1 revealed seminiferous tubule degeneration, sloughing of germ cells, and multinucleated symplasts (Figures 4F and 4H; black arrows), findings also described in cyclin A1 (*Ccna1*) knockout mice, which are infertile due to a spermatocyte block (Liu et al., 1998). Thus, JQ1 treatment causes a block in spermatogenesis and a resultant reduction in testicular production of spermatozoa.

Because absence of glycoprotein hormone and abrogation of androgen signaling pathways cause male infertility (Matzuk and Lamb, 2008), we measured the serum levels of follicle-stimulating hormone (FSH), luteinizing hormone (LH), and testosterone after treatment with JQ1. Differences in the levels of FSH, LH, and testosterone were not statistically significant between control and JQ1-treated males (Figures 3K–3M). Likewise, weights of the seminal vesicles, a major androgen-responsive tissue, were similar (Figure S1). These results are consistent with the presence of histologically normal testosterone-producing Leydig cells in JQ1-treated males (Figures 4A and 4B; red arrows). Thus, JQ1 effects are specific to germ cells and do not alter hormone-dependent processes.

### Molecular Analysis of the Germ Cell Defects in JQ1-Treated Testes

**Figure 5:**
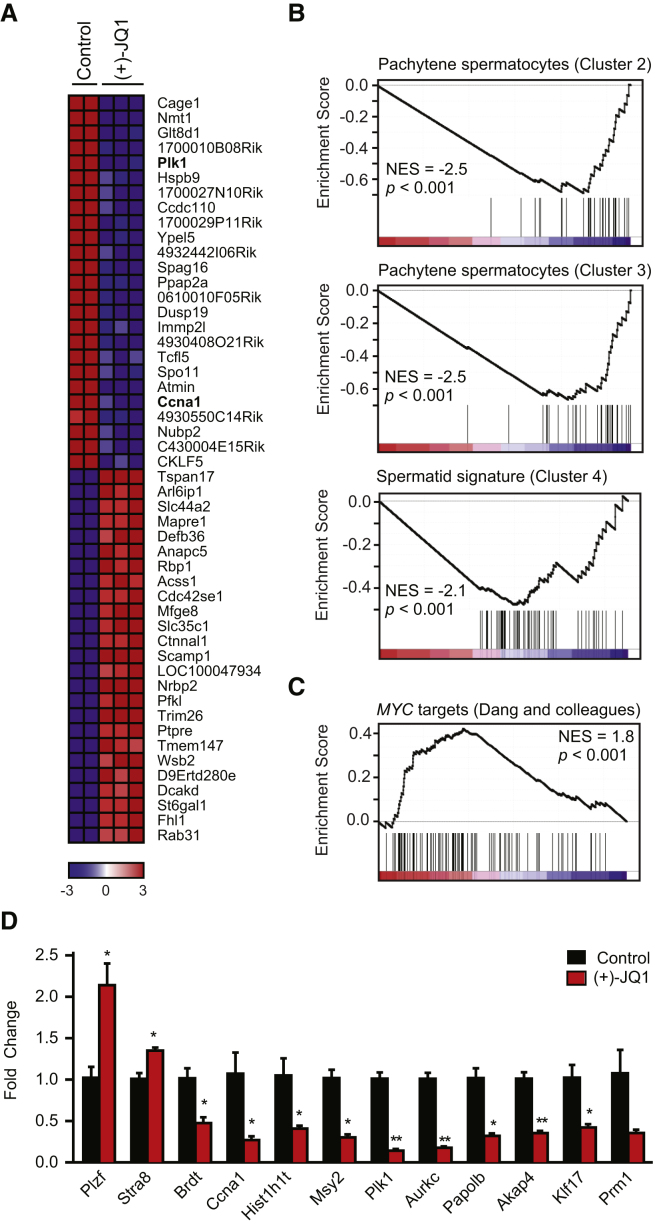
Selective Depletion of Germ Cell Transcripts by JQ1 (A) Heatmap representation of the top 25 down- and upregulated genes (Q < 0.05) following treatment of male mice with JQ1 (50 mg/kg from 6–12 weeks). *Plk1* and *Ccna1* (bold) are downregulated by JQ1 exposure. (B) GSEA of three spermatogenesis gene clusters (Schultz et al., 2003) in the testicular transcriptional profile of JQ1-treated males. (C) GSEA of a canonical MYC-dependent gene set (Zeller et al., 2003) in the testicular transcriptional profile of JQ1-treated male mice. (D) Quantitative RT-PCR analysis of males treated from 6–12 weeks of age with JQ1 or vehicle. The mouse genes are *Plzf* (promyelocytic leukemia zinc-finger or *Zbtb16*), *Stra8* (stimulated by retinoic acid gene 8), *Brdt* (bromodomain, testis-specific), *Ccna1*, *Hist1h1t* (histone cluster 1, histone 1, testis-specific), *Msy2* (Y box protein 2 or Ybx2), *Papolb* (poly (A) polymerase β or Tpap), *Klf17* (Kruppel-like factor 17 or Zfp393), and *Prm1* (protamine 1). Data represent the mean ± SEM and are annotated with p values as obtained from a two-tailed t test (^∗^p < 0.05; ^∗∗^p < 0.001; the p value for *Prm1* is 0.06). See also Tables S5 and S6.

To molecularly define the consequences of BRDT inhibition by JQ1, we performed genome-wide expression analysis of males treated with 50 mg/kg JQ1 daily for 6 weeks (6–12 weeks of age). Hierarchical clustering separated samples by treatment assignment and identified 1,685 unique genes enriched or upregulated and 675 unique genes depleted or downregulated (2-fold or greater; Q value < 0.05) with JQ1 exposure (Figure 5A). The broad transcriptional events observed—more than an order of magnitude greater than prior studies of JQ1 (Delmore et al., 2011) —suggested a pronounced effect on multistage differentiation more than selective effects on discrete transcriptional programs. Multiple germ-cell-expressed genes were suppressed upon treatment with JQ1, including *Ccna1* (expressed in pachytene spermatocytes), *Msy2* (pachytene spermatocytes and postmeiotic germ cells), polo-like kinase 1 (*Plk1*; diplotene and diakinesis stage spermatocytes, secondary spermatocytes, and round spermatids), aurora kinase C (*Aurkc*; chromocenter clusters in diplotene spermatocytes), and A-kinase anchoring protein 4 (*Akap4*; postmeiotic germ cells). These results are significant because knockout mice lacking cyclin A1 have a spermatocyte block (Liu et al., 1998); *Msy2* knockout mice are sterile due to postmeiotic germ cell block and multinucleated spermatids (Yang et al., 2005); absence of AKAP4 prevents progressive motility (Miki et al., 2002); humans with aurora kinase C mutations demonstrate polyploid nuclei secondary to meiosis I arrest (Dieterich et al., 2007); and the mammalian PLK1 protein, similar to fruit fly polo kinase, is believed to function in the completion of meiotic division.

To determine the stages of spermatogenesis at which JQ1 functions, we then queried these data with functionally defined gene sets reflecting early (pachytene spermatocyte) or late (spermatid) transcriptional signatures (Schultz et al., 2003) and observed coordinate depletion (normalized enrichment score [NES] = −2.5 to −2.1; p < 0.001) in three independent modules by gene set enrichment analysis (GSEA).

Our expression profiling results were confirmed and extended by gene-specific RT-PCR (Figure 5D and Table S6). Genes expressed early in spermatogenesis such as *Plzf*, a spermatogonial marker, and *Stra8*, expressed in differentiating spermatogonia and preleptotene spermatocytes, are 2.0- and 1.3-fold enriched, respectively, in testes of JQ1-treated mice compared to controls. In addition to *Ccna1*, *Msy2*, *Plk1*, *Aurkc*, and *Akap4*, other key genes expressed during meiosis or spermiogenesis, including *Brdt* (mid- to late-spermatocytes), *Papolb* (step 1–7 round spermatids), *Klf17* (step 4–7 spermatids), and *Prm1* (step 7–16 spermatids), are 2.1- to 7.3-fold lower in testes of mice treated with JQ1 versus control. Unlike the *Brdt* mutant mouse (Shang et al., 2007), in which pachytene spermatocyte-expressed gene *Hist1h1t* is upregulated, JQ1 treatment leads to a 2.6-fold downregulation of this gene, in line with suppression of *Brdt*, *Ccna1*, *Msy2*, and *Plk1*. This difference is likely secondary to enhanced blockade of BRDT function by JQ1 in spermatocytes compared to the *Brdt* hypomorphic mouse.

**Figure 6:**
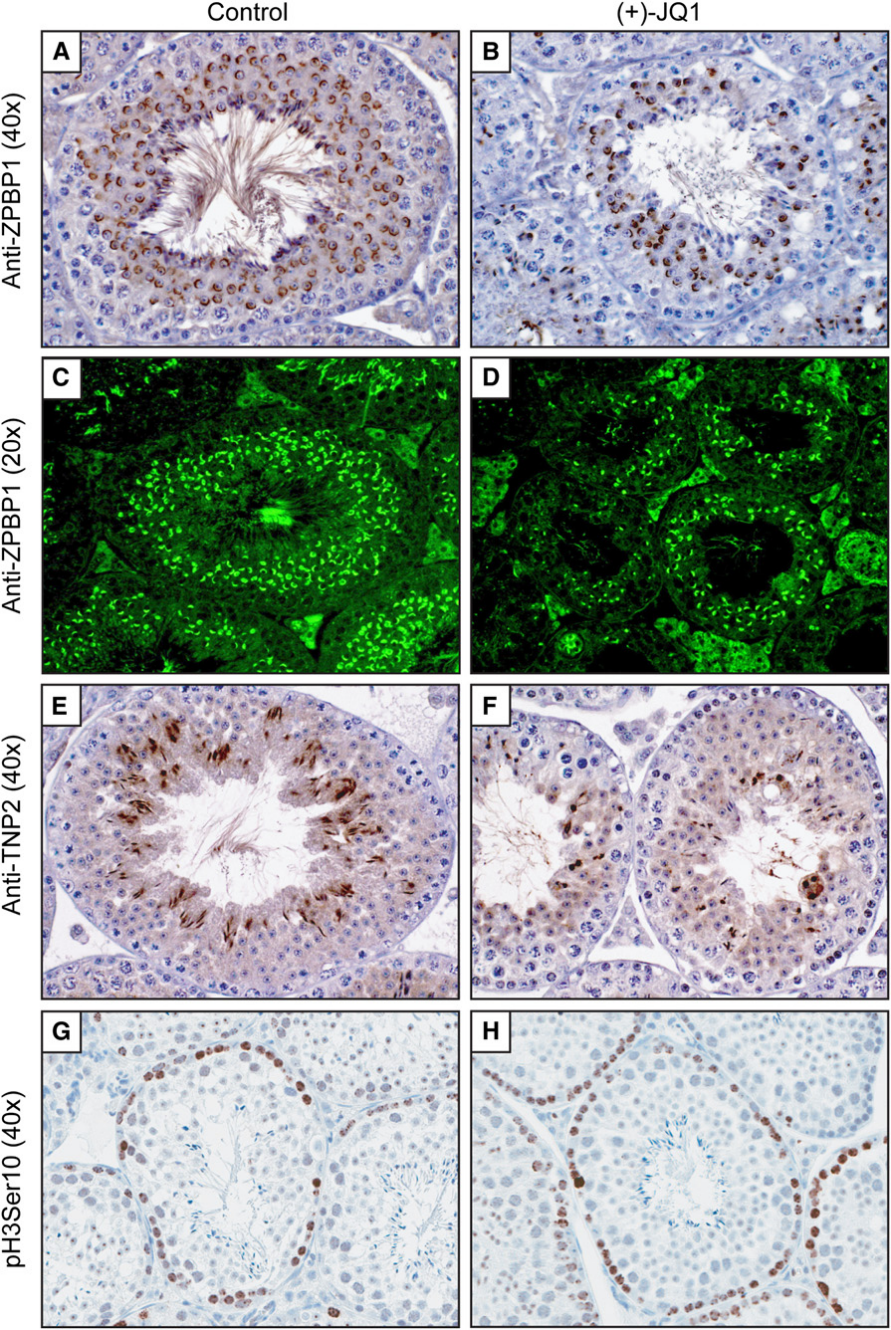
Histological Evidence of Antispermatogenic Effects of JQ1 (A and B) Immunohistochemical analysis (40×) of ZPBP1 in stage VII seminiferous tubules of males treated daily from 6–12 weeks with (A) vehicle or (B) JQ1. (C and D) Immunofluorescence analysis (20×) of ZPBP1 in testis tubules of males treated BID from 9–12 weeks with (C) vehicle or (D) JQ1. (E and F) Immunohistochemical analysis (40×) of TNP2 in testis tubules of males treated BID from 9–12 weeks with (E) vehicle or (F) JQ1. (G and H) Exposure to JQ1 has no effect on mitotic progression or meiotic chromatin condensation, as determined by histone 3 phosphoserine 10 (pH3Ser10) staining of testis tubules of males treated daily from 6–12 weeks with (G) vehicle or (H) JQ1. See also Figures S2 and S3.

**Figure S2:**
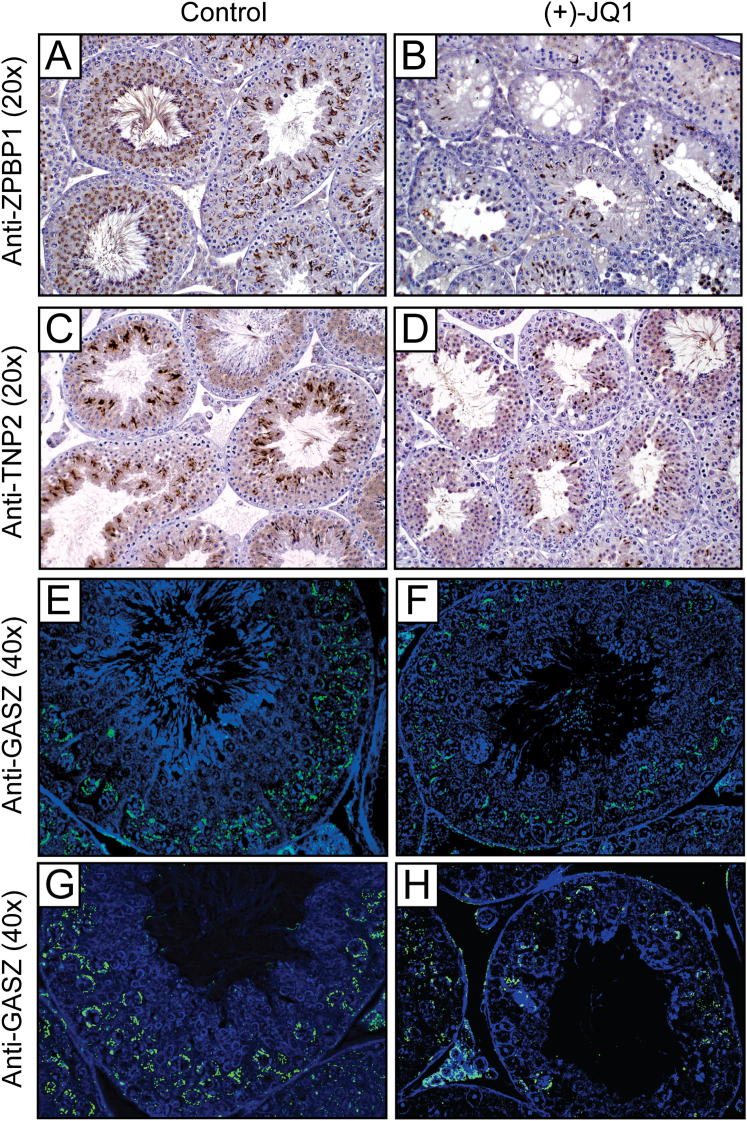
Histological Characterization of the Testes of Mice Treated with JQ1 or Control, Related to Figure 6 (A and B) Immunohistochemical study (20x) of ZPBP1 in seminiferous tubules of male mice treated daily from 6-12 weeks with vehicle control (A) or JQ1 (B). (C and D) Immunohistochemical study (20x) of TNP2 in testis tubules of male mice treated BID from 9-12 weeks with vehicle control (C) or JQ1 (D). (E–H) Immunofluorescence study (40x) of GASZ in testis tubules of male mice treated BID from 9-12 weeks with vehicle control (E, G) or JQ1 (F, H). GASZ is expressed in nuage granules in pachytene spermatocytes.

To further characterize the consequences of these molecular changes, we performed histological analysis of zona pellucida binding protein 1 (ZPBP1) and transition protein 2 (TNP2). ZPBP1 is an acrosomal matrix protein first detected in round spermatids as the acrosome forms (Lin et al., 2007), and TNP2 is expressed in the nuclei of step 10–15 spermatids during histone-to-protamine transition (Zhao et al., 2004). Consistent with transcriptional analysis, JQ1 treatment for 3 or 6 weeks reduced the number of round spermatids, elongating spermatids, elongated spermatids, and spermatozoa that are positive for ZPBP1 (Figures 6A–6D and S2) and TNP2 (Figures 6E and 6F and S2). Depending on the severity of the cellular loss in any specific tubule, JQ1 treatment caused variable losses of spermatocytes positive for GASZ (Figure S2), a piRNA pathway protein expressed in pachytene spermatocytes in adults (Ma et al., 2009). The significant effect of JQ1 on the seminiferous tubule compartment confirms the transfer of JQ1 across the blood:testis boundary to alter spermatogenesis.

**Figure S3:**
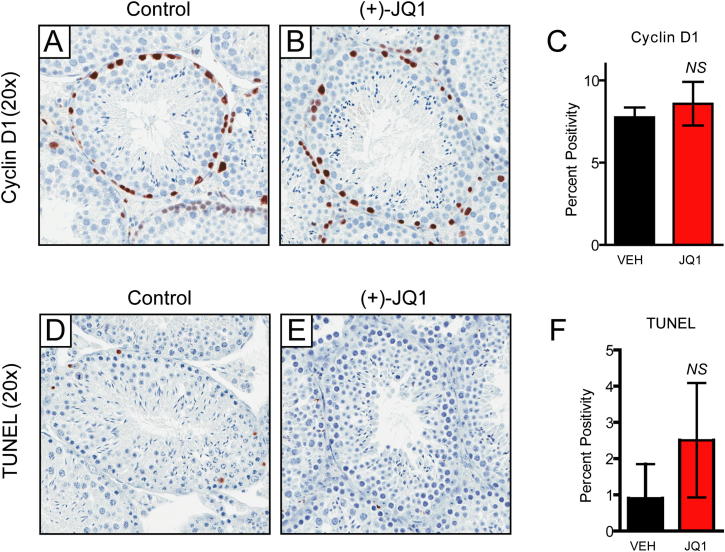
BRDT Inhibition by JQ1 Does Not Affect Proliferating Gonocytes and Spermatogonia, Related to Figure 6 (A–C) (A and B) Staining of Cyclin D1 in the epididymides of male mice treated with control or JQ1 from 6-12 weeks of age reveals no effect on the number of stained spermatogonia by quantitative immunohistochemistry as shown in (C). Data represented as mean ± SEM. (D–F) (D and E) JQ1 treatment does not elicit a significant apoptotic response in seminiferous tubules of male mice, compared to vehicle-treated animals, as determined by TUNEL staining and quantitative immunohistochemistry as shown in (F). Data represented as mean ± SEM.

The Bradner laboratory has demonstrated a pronounced antimitotic effect of JQ1 on dividing cancer cells (Delmore et al., 2011; Filippakopoulos et al., 2010). In BRD4-dependent cancers, the antitumor efficacy of JQ1 is associated with G1 cell-cycle arrest. Toxicity to proliferative compartments such as bone marrow or bowel has not been observed. To firmly exclude a BRD4-mediated, nonspecific antimitotic effect of JQ1 in testes, we stained testis sections for phosphorylated serine 10 of histone H3 (pH3Ser10), which accumulates in the nuclei of mitotic spermatogonia during chromatin condensation. Changes in pH3Ser10 are not seen, supporting a selective effect of JQ1 on spermatocytes and during spermiogenesis (Figures 6G and 6H). Further supporting a nontoxic effect of JQ1 on spermatogonia, quantitative microscopic analysis failed to identify any statistically significant decrease in the abundance of cyclin-D1-positive nuclei or any increase in TUNEL-positive cells (Figure S3).

In translational models of solid and hematologic malignancies, the cancer-specific antiproliferative activity of JQ1 has been mechanistically linked to addiction to the *BRD4* proto-oncogene (Filippakopoulos et al., 2010) or oncogenic *MYC* (a BRD4 target gene) (Delmore et al., 2011; Zuber et al., 2011). In cancer models, BET bromodomain inhibition by JQ1 results in the coordinated downregulation of the Myc transcriptional program (Delmore et al., 2011; Zuber et al., 2011). Though *Myc* is expressed in type B spermatogonia and early prophase spermatocytes (Wolfes et al., 1989), JQ1 exposure did not suppress the expression of MYC target genes, as assessed by GSEA using the functionally defined MYC signature validated by Dang and colleagues (Figure 5C) (Zeller et al., 2003). Paradoxically, MYC-dependent genes trended toward increased expression (NES = 1.8; p < 0.001), possibly due to enrichment of spermatogonial RNA amidst depletion of spermatids and spermatozoa. Together, these orthogonal measurements of spermatogonial transcription, appearance, and function rule out a nonspecific antiproliferative effect of JQ1 on BET bromodomains within mitotic germ cells.

### BRDT Inhibition Confers a Reversible Contraceptive Effect in Males

To determine the effects of alternative JQ1 treatment regimens on the fertility of male mice, JQ1 (50 mg/kg/day) and vehicle control were each delivered to 10 male mice for 6 weeks. To ensure that JQ1 and vehicle were having proper effects, three males from each group were sacrificed and were shown to have reduced testis size (control: 107.4 ± 8.8; JQ1: 62.8 ± 7.5 mg; p < 0.05), sperm counts (control: 12.3 ± 0.6 × 10^6^; JQ1: 3.8 ± 0.3 × 10^6^; p < 0.0001), and sperm motility (control: 72.4 ± 2.5%; JQ1: 16.0 ± 0.1%; p < 0.0001). After this pretreatment period, the remaining seven JQ1-treated and seven control males were mated continuously to two adult females per male while continuing to receive JQ1 or vehicle. After the first month of breeding, the seven control males had sired 14 litters of offspring. However, only four of the seven JQ1-treated males sired offspring (only six litters of offspring and a reduced number of pups). For the three JQ1-treated males that had not yet sired offspring, these mice demonstrated normal mating behavior as evident by the presence of copulation plugs. Thus, even at a low dose of JQ1, there is a partial contraceptive effect.

**Figure 7:**
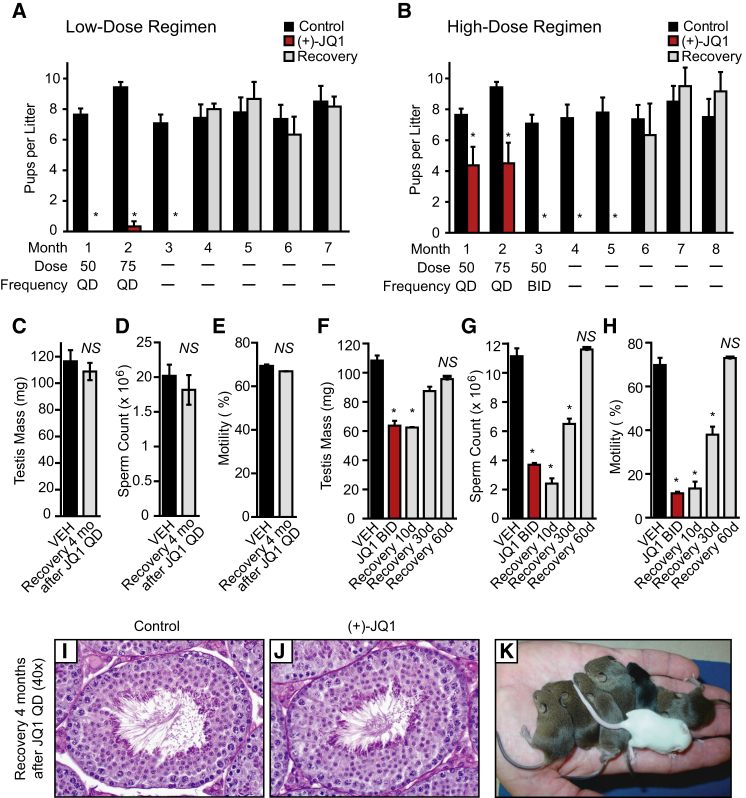
BRDT Inhibition with JQ1 Causes a Reversible Contraceptive Effect in Male Mice (A) Adult males were pretreated for 6 weeks with vehicle control (n = 7) or JQ1 (50 mg/kg QD; n = 3) and then caged continuously with two females each while continuing 50 mpk QD for month 1 and escalating to 75 mpk QD for month 2. JQ1 treatment was stopped at the end of month 2 of mating. Graphical representation of pups born in each month to the females reveals a contraceptive effect evident in months 1–3 (data represent mean ± SEM; ^∗^p < 0.001) and durable restoration of fertility at month 4. (B) Adult males were pretreated for 6 weeks with vehicle (n = 7) or JQ1 (50 mg/kg QD; n = 4) and then caged continuously with two females each while continuing 50 mg/kg QD for month 1, escalating to 75 mg/kg QD for month 2, and further escalating to 50 mg/kg BID for month 3. JQ1 treatment was stopped at the end of month 3 of mating. Graphical representation of pups born in each month to the caged females reveals a complete contraceptive effect evident in months 3–5 (data represent mean ± SEM; ^∗^p < 0.001) and durable restoration of fertility at month 6. (C–E) Graphical representation of (C) testis mass (mg), (D) sperm count, and (E) sperm motility of adult control males (n = 3) and JQ1-treated males (n = 3) following a 4 month recovery from the low-dose regimen shown in (A) (data represent mean ± SEM; NS, not significant). (F–H) Graphical representation of (F) testis mass, (G) sperm count, and (H) sperm motility of adult control males (n = 4) and JQ1-treated males after 3 weeks of 50 mg/kg BID treatment (n = 2), as well as following 10 days (n = 2), 30 days (n = 2), and 60 days (n = 2) of recovery from the high-dose (B) regimen (data represent mean ± SEM; ^∗^p < 0.05). (I and J) Microscopic analysis of seminiferous tubules from (I) control and (J) JQ1-treated males following 4 months of recovery reveals compete histological recovery. (K) Litter sizes and pups born following JQ1 cessation are normal. See also Figure S4.

As the mice appeared to be healthy at 50 mg/kg/day of JQ1, we escalated the dosage of JQ1 in the second mating month to 75 mg/kg/day. The male mice appeared to fall into two groups: those that show a contraceptive effect with 50 mg/kg/day followed by 75 mg/kg/day (Figure 7A) and those that still produced at least one litter under this lower-dose regimen (Figure 7B). For the three mice that were most responsive to the low-dose regimen, only one was able to sire a litter of two offspring (born on the first day of month 2). For the latter group, the four males that sired offspring were treated in mating month 3 with 50 mg/kg twice per day. These males were not observed to sire any offspring in month 3 (Figure 7B). Thus, depending on individual variability of each of the males, total daily dosages between 50 and 100 mg/kg can produce a complete contraceptive effect in male mice.

**Figure S4:**
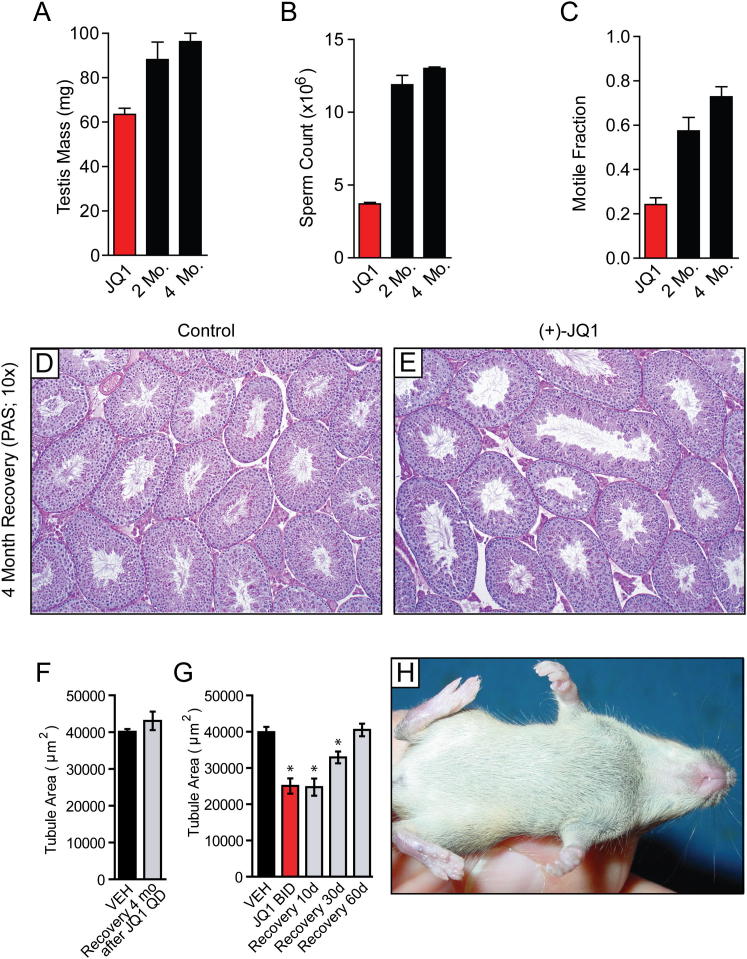
Reversal of Testicular Phenotypes following Withdrawal of JQ1, Related to Figure 7 (A–C) Analysis of (A) testicular mass, (B) sperm counts, and (C) sperm motility of adult male mice treated for 3 weeks with JQ1 (50 mg/kg QD), followed by euthanasia at the end of treatment (JQ1), 2 months following cessation of treatment, and 4 months following cessation of treatment. Normalization of all parameters is evident following drug withdrawal. Data represent mean ± SEM. (D and E) Testis histology (10X) of tubules from control and JQ1-treated male mice 4 months following cessation of JQ1 as treated in (A-C). (F) Graphical representation of tubule area calculated from histological preparations of testis tubules from male mice following four month recovery from the low-dose (Figure 6A) regimen. Data represent mean ± SEM. (G) Graphical representation of tubule area calculated from histological preparations of testis tubules from male mice during and following 10 day, 30 day, and 60 day recovery from a 3 week period of JQ1 BID treatment. Data represent mean ± SEM (^∗^, significant at p < 0.05). (H) A single pup born from a JQ1-treated male after halting JQ1 treatment. Normal developmental and behavioral phenotypes are observed in offspring of mice with restored fertility following a period of contraception conferred by JQ1 treatment.

To assess the reversibility of the JQ1-induced contraceptive effect, we examined whether fertility returned after JQ1 treatment was halted. Among the three infertile mice treated with the low-dose regimen, infertility remained complete at 1 month of recovery (mating month 3), indicating a durable effect of JQ1 on spermatogenesis. However, in mating month 4, all three males sired offspring with a statistically similar number of pups per female as observed for the controls (Figure 7A). The average days to effective copulation was estimated to be 31.7 ± 6.0 days (range: 26–38 days) based on the birth of the first litters after treatment was stopped and the 19 day gestation period of mice. After a total mating period of 7 months (i.e., 4 months after halting the low-dose regimen), testis volume, seminiferous tubule area, testis histology, sperm motility, and sperm counts were statistically similar to the control group (Figures 7C–7E and S4), consistent with fully recovered fertility. Thus, mice treated with JQ1 at 50 mg/kg/day and then 75 mg/kg/day recover fertility within 6 weeks of withholding the drug and show no long-term effects of JQ1 treatment. For the mice that required escalation to the high-dose regimen (50 mg/kg BID), JQ1 was halted after the first month of this treatment (i.e., treatment month 3). In months 4 and 5, these males failed to sire offspring (Figure 7B). However, all JQ1-treated males sired offspring in month 6 and sired similar litter sizes as controls in months 6–8. The average days to effective copulation for this cohort was estimated to be 65.7 ± 7.7 days (range: 58–81 days). Thus, longer JQ1 treatment and/or the increased dosage of JQ1 resulted in an extended (∼1 month) period of infertility.

To more precisely determine the integrity and function of the testes and spermatozoa following withdrawal of JQ1 therapy, two additional independent experiments were performed. In the first experiment, JQ1-treated adult male mice were examined at the end of 6 weeks of treatment (50 mg/kg/day) or after 2 or 4 months following cessation of therapy. After 2 months, the testis weight and sperm counts and motility were increased, and after 4 months, the weights and sperm parameters approached normal levels (Figure S4). Histologically, seminiferous tubules of JQ1-treated mice are indistinguishable from controls (Figures 7I and 7J). In the second experiment, a cohort of mice was treated with vehicle or 3 weeks of high-dose JQ1 (50 mg/kg BID). Mice were sacrificed at 3 weeks of treatment and 10, 30, and 60 days after JQ1 withdrawal. Males treated with high-dose JQ1 continue to show defects in testicular parameters 30 days after halting treatment but exhibit complete recovery of all parameters within 60 days (Figures 7F–7H), consistent with the documented recovery of fertility. Thus, depending on duration and overall dosage of JQ1, testicular and semen parameters normalize within 1–3 months to allow productive copulation and return of fertility.

Lastly, offspring that were born from JQ1-treated males showed normal size, activity, and behavior as offspring born from controls (Figure 7K and data not shown). When offspring from vehicle and JQ1-treated fathers were allowed to mate with adult females (2 females/cage) at 6 weeks of age, both sets of males (n = 6 for each group) displayed normal mating behavior, sired offspring from each female, and produced statistically similar litter sizes from matings over the first month (control: 7.73 ± 0.70 pups/litter; JQ1: 7.50 ± 0.34 pups/litter). At 11 weeks of age, the testis weights (control: 98.7 ± 2.8 mg; JQ1: 95.8 ± 3.2 mg), sperm counts (control: 11.8 ± 0.3 × 10^6^; JQ1: 11.2 ± 0.6 × 10^6^), and sperm motility (control: 69.5 ± 1.7%; JQ1: 69.8 ± 1.3%) of the offspring were statistically similar. Together, these findings indicate that JQ1 did not have any long-term transgenerational effects on testis physiology or reproductive capacity.

## Discussion

Using a highly potent and selective chemical probe, our studies provide pharmacologic validation of the amino-terminal bromodomain of BRDT as a target for male contraception. JQ1 emerges as a lead compound for a new class of drugs that can cross the blood:testis boundary, inhibit bromodomain activity during spermatogenesis, impair sperm generation and motility, and produce a reversible contraceptive effect in mammals. These data support JQ1 as the first contraceptive agent that selectively and reversibly targets the male germ cell.

Modulation of chromatin structure during spermatogenesis by an inhibitor of an epigenetic reader protein, such as BRDT, raises concern for epigenetic developmental abnormalities in progeny born to animals on treatment or following cessation. To date, we have not identified any anomalous phenotypes in offspring during or following exposure to JQ1. Targeting the male germ cell might raise concern for irreversible toxicity and resultant infertility. We have only observed full recovery of fertility in treated males; testis size, seminiferous tubule diameter, histological appearance, sperm number and motility, and fertility of JQ1-treated males recover to the levels of controls (Figures 7 and S4). These observations are consistent with the restricted expression of the BRDT target in pachytene spermatocytes through round spermatids and with the function of BRDT in these stages to promote spermatogenesis. Indeed, the phenotype of mice treated with JQ1 closely resembles the seminal findings by Wolgemuth and colleagues in the characterization of BRDT(1) hypomorphic mice (Shang et al., 2007). We cannot rule out that some of effects of JQ1 are through inhibition of other members of the BET subfamily that are also expressed in murine testes (e.g., BRD4 [mRNA expressed in spermatogonia], BRD2 [diplotene spermatocytes], and BRD3 [round spermatids]) (Shang et al., 2004). However, the mitotic status of spermatogonia is unaltered in the JQ1-treated males, and we do not observe end-organ toxicity in testis or other tissues in mice, such as the gastrointestinal mucosa, suggestive of a generalized antimitotic effect, as might be expected with inhibition of BRD4 (Dey et al., 2003). Unequivocally, JQ1 is a BET family inhibitor that exerts its contraceptive effect on the male germline during the peak of expression of BRDT.

**Figure S5:**
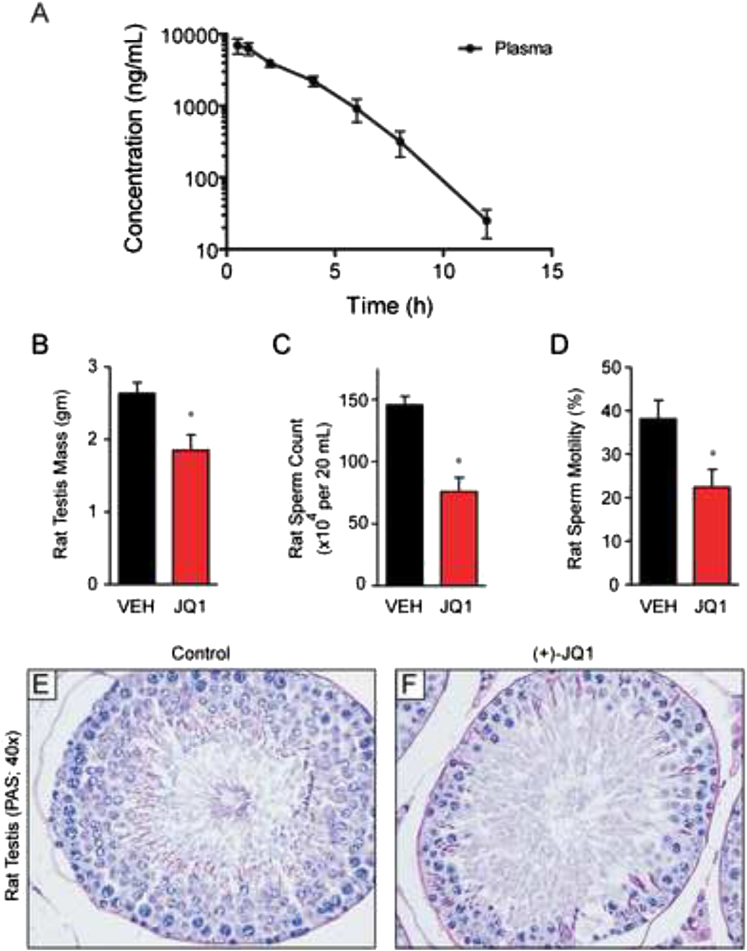
JQ1 Reduces Testis Size, Sperm Counts, and Motility in Male Rats, Accompanied by Histological Evidence of Impaired Spermiogenesis, Related to Figure 4 (A) Pharmacokinetic analysis of JQ1 in the plasma of male rats following a single administration of JQ1 (50 mg/kg IP). Data represent the mean ± SD. (B–D) Graphical representation of (B) testis mass, (C) sperm count and (D) sperm motility derived from adult male Sprague-Dawley rats treated with intraperitoneal JQ1 for 3 weeks, as described in the Experimental Procedures. Reduction of each parameter is consistent with an anti-spermatogenic effect of BRDT bromodomain inhibition by JQ1. Data represent the mean ± SEM (^∗^, significant at p < 0.05). (E and F) Testis histology (40x) of seminiferous tubules from control and JQ1-treated male rats. Comparable depletion of maturing spermatocytes and round spermatids is evident in treated Sprague-Dawley rats compared to male mice (as shown in Figure 4).

This chemical genetic study of bromodomain inhibition for male contraception provides pharmacologic target validation, encourages further drug development efforts, and offers insights guiding and enabling pharmaceutical BRDT inhibitor development. As this index study closely examines effects on human and murine BRDT, further study of additional species may be relevant for drug development. Toward this objective, we have completed preliminary studies of JQ1 in rats in which we have observed excellent pharmacologic exposure to JQ1 by i.p. injection (Figure S5A and Table S7) and have identified a tolerated dose (10 mg/kg BID), which is effective in reducing testis mass, sperm count, and motility (Figures S5B–S5D), accompanied by comparable histological evidence of impaired spermiogenesis (Figure S5E). For a chemical probe intended for laboratory research, JQ1 has excellent drug-like properties yet possesses a short terminal elimination half-life. Pharmacokinetic studies performed herein demonstrate that excellent drug exposure can be accomplished with twice-daily administration. The observed complete contraceptive effect of JQ1 when given twice daily suggests that more continuous inhibition of BRDT-dependent effects on spermatogenesis may be required for clinical translation of this research.

We are currently creating and testing the effects of JQ1 derivatives possessing selectivity for BRDT to extend the favorable therapeutic window of BRDT inhibition for male contraception. Recently described tool compounds of BET bromodomains provide divergent putative scaffolds for lead optimization (Chung et al., 2011; Dawson et al., 2011; Nicodeme et al., 2010). Compounds that demonstrate higher affinity and specificity for BRDT would be expected to reduce any possible long-term, adverse effects of a pan-BET bromodomain inhibitor. Structural and sequence comparisons of BRDT(1) and BRD4(1) reveal differences in surface residues that may be explored to develop further isoform-selective BET inhibitors. Because human and mouse BRDT proteins are highly conserved and have nearly identical bromodomain pockets based on our structural predictions, we envision that our discoveries can be completely translated to men, providing a novel and efficacious strategy for a male contraceptive.

## Experimental Procedures

### (+)-JQ1

The direct-acting, small-molecule bromodomain inhibitor was prepared as described (Filippakopoulos et al., 2010).

### Protein Cloning

cDNA-encoding BRDT (human: NCBI accession number AAB87862.1, obtained from FivePrime; murine: NCBI accession number EDL20168.1, obtained from Dr. Saadi Khochbin) was used as template to PCR amplify the N-terminal bromodomain regions of human and murine BRDT. PCR products were isolated and subcloned into a pMCSG7-derived expression vector (pNIC28-Bsa4), using ligation-independent cloning. The constructs (hBRDT(1) 21–137, hBRDT(2) 257–382, mBRDT(1) 23–133, and mBRDT(2) 257–383) were transformed into competent Mach1 cells (Invitrogen, UK) to yield the final plasmid.

### BRDT Proximity Assay

AlphaScreen assays were performed on an Envision 2104 instrument with minor modifications from the manufacturer’s protocol (PerkinElmer, USA).

### Isothermal Titration Calorimetry

Isothermal titration calorimetry (ITC) was carried out on a VP-ITC titration microcalorimeter from MicroCal, LLC (Northampton, MA). Dissociation constants and thermodynamic parameters are listed in Figure 1.

### Crystallization of BRDT and (+)-JQ1

BRDT(1) crystallizations were carried out using the sitting drop vapor diffusion method at 4°C using a mosquito crystallization robot (TTP Labtech, Royston, UK). Data collection and refinement statistics can be found in Table S2. The model and structure factors have been deposited with PDB accession code: 4FLP.

### Mouse Contraceptive Studies

(+)-JQ1 was dissolved in DMSO at 50 mg/ml, 75 mg/ml, or 100 mg/ml and then diluted 1:10 in 10% (2-Hydroxypropyl)-β-cyclodextrin (Sigma-Aldrich) or 20% Captisol (CYDEX, Lenexa, KS). The mixture was injected i.p. into male mice at 1% of the body weight (50 mg/kg or 75 mg/kg) or 0.5% of the body weight for the 50 mg/kg BID injections. The control was DMSO dissolved 1:10 in 10% (2-Hydroxypropyl)-β-cyclodextrin or 20% Captisol and injected similarly. Juvenile or adult C57BL6/J/129S5 hybrid mice for these studies were weighed daily before injections and fed ad libitum.

For mating studies, males were pretreated for 6 weeks with vehicle or JQ1 and caged continuously with two females each while continuing 50 mpk QD for month 1 and escalating to 75 mg QD for month 2. The JQ1-treated males that were responsive to this “low-dose” treatment regimen were allowed to recover untreated while continuing to breed to 2 females per cage through 7 months of continuous breeding. Males that continued to sire offspring in month 2 were treated in month 3 of breeding with 50 mg/kg BID (“high-dose” regimen), and JQ1 treatment was stopped for these males and controls while continuing to breed to 2 females per cage through 8 months of continuous breeding. Cages were checked daily, and offspring born per each female were recorded.

For the recovery studies, males were sacrificed post-JQ1 and control treatment, and testis and sperm parameters were determined. These studies were approved by the Administrative Committee on Laboratory Animal Care at Baylor College of Medicine, and all experiments were conducted in accordance with the NIH guide for the Care and Use of Laboratory Animals.

### Histology and Measurement of Testis Seminiferous Tubule Area

Testes and epididymides were fixed in Bouin’s solution and processed for sectioning. Digital images of cross-sections were captured, and 25 tubules were randomly selected for each sample. Seminiferous tubule area was determined using NIH Image J software. The mean ± SEM of the tubule area for each treatment group was calculated, and comparisons between control and JQ1 treatment groups were made using a t test.

### Epididymal Sperm Counts and Motility

Counts were performed on spermatozoa isolated from the entire epididymis or from the caudal epididymis of mice as described (Roy et al., 2007). In brief, epididymides were dissected and placed in prewarmed M2 medium, minced, and incubated at 37°C for 30 min in a CO_2_ incubator prior to counting and analysis of sperm motility.

### Gene Expression Analyses

Total RNA from mouse testes was isolated using TRIzol reagent (Invitrogen) and processed for Illumina gene expression analysis or QPCR.

Extended Experimental Procedures **Protein Expression and Purification**

Colonies from freshly transformed plasmid DNA in competent *E. coli* BL21(DE3)-R3-pRARE2 cells (phage-resistant derivative of BL21(DE3) cell [Invitrogen] with a pRARE-plasmid-encoding rare codon tRNA) were grown overnight at 37°C in 5 ml of Luria-Bertani medium (LB-broth, Merck) with 50 μg/ml kanamycin and 34 μg/ml chloramphenicol (startup culture). The startup culture was diluted 1:1,000 in fresh medium, and cell growth continued at 37°C to an optical density of about 0.3 (OD600) before the temperature was decreased to 18°C. When the system equilibrated at 18°C and the optical density was about 0.6 (OD600), protein expression was induced overnight at 18°C with 0.1 mM isopropyl-β-D-thiogalactopyranoside (IPTG). The bacteria were harvested by centrifugation (8,700 × *g* for 15 min at 4°C, JLA 81,000 rotor, on a Beckman Coulter Avanti J-20 XP centrifuge) and were frozen at −20°C as pellets for storage. Cells expressing His6-tagged protein were resuspended in lysis buffer (50 mM HEPES [pH 7.5] at 25°C, 500 mM NaCl, 10 mM Imidazole, 0.5 mM TCEP) in the presence of protease inhibitor cocktail (1 μl/ml) and were lysed using an EmulsiFlex-C5 high-pressure homogenizer (Avestin [Mannheim, Germany]) at 4°C. The lysate was cleared by centrifugation (17,000 × *g* for 30 min at 4°C, JA 25.50 rotor, on a Beckman Coulter Avanti J-20 XP centrifuge) and was applied to a diethylaminoethyl cellulose column (DE52 anion exchanger, Whatman Int. Ltd, 10 gr in 100 ml of 2.5 M NaCl, equilibrated with 100 ml lysis buffer) that was serially connected to a nickel-nitriloacetic acid agarose column (Ni-NTA, QIAGEN Ltd., 5 ml, equilibrated with 20 ml lysis buffer). The column ensemble was washed with 50 ml of lysis buffer, and the DE52 column was discarded. The Ni-NTA column was washed twice with 10 ml of lysis buffer containing 30 mM imidazole, and the protein was eluted using a step elution of imidazole in lysis buffer (50, 100, 150, 2 × 250 mM imidazole in 50 mM HEPES [pH 7.5] at 25°C, 500 mM NaCl). All fractions were collected and monitored by SDS-polyacrylamide gel electrophoresis (Bio-Rad Criterion Precast Gels, 10%–20% Tris-HCl 1.0 mm, from Bio-Rad, CA; Gel electrophoresis conditions: 180 V, 400 mA, 55 min in SDS buffer). After the addition of 10 mM dithiothreitol (DTT), the eluted protein was treated overnight at 4°C with TEV protease to remove the hexa-histidine tag. The proteins were further purified with size exclusion chromatography on a Superdex 75 16/60 HiLoad gel filtration column (GE/Amersham Biosciences) on an ÄktaPrime plus system (GE/Amersham Biosciences). Samples were monitored by SDS-polyacrylamide gel electrophoresis, concentrated to 10–40 mg/ml in the elution buffer, 10 mM HEPES (pH 7.5), 150 mM NaCl, and 0.5 mM TCEP and were used for crystallization. Protein handling was carried out on ice or in a cold room in all of the above steps.

**BRDT Proximity Assay**

For the AlphaScreen assays, all reagents were diluted in 50 mM HEPES, 150 mM NaCl, 0.1% w/v BSA, and 0.01% w/v Tween20 (pH 7.5) and were allowed to equilibrate to room temperature prior to addition to plates. After addition of Alpha beads to master solutions, all subsequent steps were performed in low-light conditions. A 2× solution of components with final concentrations of BRDT at 80 nM, Ni-coated Acceptor Bead at 25 μg/ml, and 80 nM biotinylated H4-tetra acetyl was added in 10 μl to 384-well plates (AlphaPlate - 384, PerkinElmer, USA). Biotinylated peptide for BRDT was synthesized in-house on a CEM Liberty 9008005 microwave peptide synthesizer: H4-tetra acetyl, Biotin-PEG2-SGRGKacGGKacGLGKacGGAKacRHRK-COOH. Addition to wells was performed with either a multichannel pipet (for optimization experiments) or a Biotek EL406 liquid handler. After a 1 min 1000 rpm spin-down, 100 nl of compounds from stock plates were added by pin transfer using a Janus Workstation (PerkinElmer, USA). The streptavidin-coated donor beads (25 μg/ml final) were added as with previous solution in a 2x, 10 μl volume. Following this addition, the plates were sealed with foil to block light exposure and to prevent evaporation. The plates were spun down again at 1000 rpm for 1 min, and the plates were incubated at room temperature for 1.5 hr prior to reading the assay.

**Isothermal Titration Calorimetry**

Isothermal titration calorimetry experiments were carried out at 10°C in 50 mM HEPES pH 7.5 (at 25°C), 150 mM NaCl. The calorimetric cell was loaded with a solution of the protein sample (54 μM protein or an equimolar mixture of 54 μM protein and (+)-JQ1, in 50 mM HEPES, pH 7.4, 150 mM NaCl). The peptide (1.5 mM in 50 mM HEPES, pH 7.4, 150 mM NaCl) was loaded into the micro-syringe. Titrations were conducted using an initial control injection of 2 μl followed by 30 identical injections of 8 μl. The collected data were corrected for peptide heat of dilution (measured on a separate experiment by titrating the peptide into 50 mM HEPES, pH 7.4, 150 mM NaCl) and normalized using the MicroCal Origin software supplied with the instrument. Thermodynamic parameters were calculated using the basic equation of thermodynamics (ΔG = ΔH - TΔS = -RTlnKB, where ΔG, ΔH and ΔS are the changes in free energy, enthalpy and entropy of binding respectively). In all cases, a single binding site model was employed, supplied with the MicroCal Origin software package.

**Crystallization of BRDT and (+)-JQ1**

BRDT(1) crystals with (+)-JQ1 (1 mM final concentration) were grown by mixing 50 nl of protein (23.2 mg/ml) with 100 nl of reservoir solution containing 1.0 M bis-tris propane pH 8.0, 25% PEG3350, 0.15 M KSCN and 10% ethylene glycol. Crystals were cryo-protected using the well solution supplemented with additional ethylene glycol and were flash frozen in liquid nitrogen. Data were collected at Diamond on beamline I03 using an ADSC Q315 detector at 0.9763 Å. Indexing and integration was carried out using MOSFLM and scaling was performed with SCALA. Initial phases were calculated by molecular replacement with PHASER (McCoy et al., 2005) using the known model of BRDT (PDB ID 2RFJ). Initial models were built by ARP/wARP (Perrakis et al., 1999) and building was completed manually with COOT (Emsley and Cowtan, 2004). Refinement was carried out in REFMAC5 (Murshudov et al., 1997). Thermal motions were analyzed using TLSMD (Painter and Merritt, 2006), and hydrogen atoms were included in late refinement cycles.

**Sequence Alignment**

Amino acid sequences for full-length bromodomain-containing proteins were obtained from the US National Heart, Lung and Blood Institute (Human BRDT accession number Q58F21; Human BRD4 accession number O60885; Mouse BRDT accession number Q91Y44). Multiple sequence alignment of full-length BRDT and BRD4 was performed using MAFFT (v6.240) (Katoh and Toh, 2008). The E-INS-i algorithm was selected as suitable for sequences containing potentially large unalignable regions, and the BLOSUM62 scoring matrix was used as suitable for highly evolutionarily conserved sequences. Gap opening penalty and offset value were set to default parameters.

**Gene Expression Analyses**

For genome-wide transcriptional analysis, background-corrected and normalized expression data for the two technical replicates were averaged before analysis. All negative values were set to 1. Differential expression was assessed using the Comparative Marker Selection module of GenePattern (Reich et al., 2006). Genes with a Q-value < 0.05 and fold change > 2 were considered significant. For GSEA, gene sets were obtained from published sources (Schultz et al., 2003; Zeller et al., 2003). In the case of the “Spermatogenic cluster” signatures derived from Schultz et al. (Schultz et al., 2003), named genes from Table S3 and updated assignments for the Unigene clusters in Table S4 (based on Unigene data downloaded April 2012) were combined. Signal-to-noise was used as the metric for GSEA (Subramanian et al., 2005). For QPCR, total RNA was reversely transcribed using Superscript III reverse transcriptase (Invitrogen). Quantitative PCR was performed using SYBR green master mix and customized primers (Table S6). All quantitative PCR assays were conducted in duplicate for each sample. *Gapdh* was used as an internal control for the quantification.

**Histological Analysis**

Histological analysis of Bouin’s fixed testes and epididymides was performed as previously described (Kumar et al., 1997) using Periodic acid-Schiff and hematoxylin. Rabbit anti-TNP2 (1:500) staining and hematoxylin counter-staining was performed as described (Zhao et al., 2004) using Bouin’s fixed testes. The goat anti-ZPBP1 (1:500) staining was performed as described (Lin et al., 2007). The guinea pig anti-GASZ (1:500) staining was performed as described (Ma et al., 2009).

**Hormone Analyses**

Blood samples were collected by cardiac puncture from mice anesthetized with isoflurane. The serum was separated from the blood and stored at –20°C until assayed. Serum FSH, LH, and testosterone levels were measured by the Ligand Assay and Analysis Core at the Center for Research in Reproduction, University of Virginia. Assay details can be found at http://www.healthsystem.virginia.edu/internet/crr/).

**Rat Spermatogenesis Study**

Adult male Sprague-Dawley rats were treated with vehicle or (+)-JQ1 (10 mg/kg formulated in saline, 10% 2-Hydroxypropyl-β-cyclodextrin and 10% DMSO). Treatment was administered IP at 1/100 body mass. Rats were checked twice-daily for mortality and weighed on days 1, 3, 7, 14, and 21. The treatment regimen utilized 4 days of 50 mg/kg JQ1 administered daily which was decreased to 10 mg/kg twice daily for the remainder of the study due to the appearance of adverse effects in a subset of animals. For all animals completing 3 weeks of treatment, testis mass, sperm motility, and sperm counts were determined as described for mouse studies. In brief, testes were fixed in Bouin’s and prepared for histology. The other half was minced in warm M16 buffer and used for sperm counts and motility studies.

## Accession Numbers

Atomic coordinates and structure factors for the reported crystal structure of BRDT(1) with JQ1 have been deposited with the Protein Data Bank under accession code 4FLP. Gene expression data were deposited in the GEO database with accession GSE37894.

## Supplemental Information

Document S1. Tables S1–S7 and Data S1 and S2

Document S2. Article plus Supplemental Information
