# Supplementary material for: Small-Molecule Inhibition of BRDT for Male Contraception
Source: Cell. 2012 Aug 17;150(4):673–84. doi: 10.1016/j.cell.2012.06.045 (PMC3420011; doi:10.1016/j.cell.2012.06.045)
Supplement: Document S1. Tables S1–S7 and Data S1 and S2 [file mmc1.pdf]

SUPPLEMENTAL TABLES AND SUPPLEMENTAL DATA

Table S1. **Isothermal Titration Calorimetry of Human BRDT(1) with a Tetra-Acetylated Histone H4 Peptide ($H4_{1\text{-}20}$K5acK8acK12acK16ac) in the Absence and Presence of (+)-JQ1, Related to Figure 1**

As two BRDT(1) proteins bind one peptide the binding constants and thermodynamic parameters are presented as sum (enthalphy) and average (Kd) of two distinct binding events.

Protein	[P] (µM)	[pep] (mM)	K_d (µM)	ΔH^{obs} (kcal/mol)	N	$T\Delta S$ (kcal/mol)	ΔG
BRDT(1)	54	1.5	25.4 ± 0.4	-7.98 ± 0.004	2.07 ± 0.008	2.02	-5.96
BRDT(1)/JQ1	54	1.5	26.0 ± 0.9	-6.07 ± 0.023	0.43 ± 0.014	0.12	-5.95

Table S2. Data Collection and Refinement Statistics, Related to Figure 2

**Data Collection**	
PDB ID	XXXX
Space group	$P2_122_1$
Cell dimensions: a, b, c (Å)	37.41 57.36 128.87
α, β, γ (deg)	90.00 90.00 90.00
Resolution* (Å)	2.20 (2.20-2.32)
Unique observations*	14655 (2082)
Completeness* (%)	99.5 (99.9)
Redundancy*	3.4 (3.6)
Rmerge*	0.110 (0.655)
I/ σI*	7.2 (2.0)
**Refinement**	
Resolution (Å)	2.20
R_{work} / R_{free} (%)	21.3/25.5
Number of atoms (protein/other/water)	1762/63/59
B-factors (Å^2) (protein/other/water)	47.42/37.04/37.56
r.m.s.d bonds (Å)	0.015
r.m.s.d angles (^{o})	1.660
Ramachadran Favored (%)	95.54
Allowed (%)	4.46
Disallowed (%)	0.00

* Values in parentheses correspond to the highest resolution shell.

Table S3. Pharmacokinetic Study of JQ1 (50 mg/kg IP Injection Daily) in C1 Male Mice, Related to Figure 3

Dose (mg/kg)	Dose (route)	Time (hr)	Plasma Mean (ng/mL)	Plasma SD	Testis Mean (ng/mL)	Testis SD	Brain Mean (ng/mL)	Brain SD
50	IP	0	BQL	NA	BQL	NA	BQL	NA
		0.033	7407	2008	15293	7346	2940	658
		0.083	11037	1545	16700	8679	8843	1358
		0.25	9663	1510	34267	17028	11967	777
		0.5	8470	1486	22267	1266	9930	2195
		1	4880	731	16997	7831	5527	1525
		2	4137	475	9723	1107	3733	270
		4	1987	673	4270	1170	1867	595
		6	463	371	837	652	305	241
		8	397	226	1083	359	281	183
		12	6.82	3.54	41.2	19.5	4.52	NA
		24	BQL	NA	BQL	NA	BQL	NA

PK parameters	Unit	Plasma	Testis	Brain
T_{max}	hr	0.0830	0.250	0.250
C_{max}	ng/mL	11000	34300	12000
Terminal $t_{1/2}$	hr	1.24	1.31	1.12
AUC_{last}	hr*ng/mL	22700	58800	22200
AUC_{INF}	hr*ng/mL	22700	58900	22200

Abbrevations: BQL (below the quantifiable limit of 1.00 ng/mL), NA (not available), SD (standard deviation), IP (i.p.).

Table S4. Pharmacokinetic Study of JQ1 (50 mg/kg IP Injection Twice-Daily) in C1 Male Mice, Related to Figure 3

**Dose** (mg/kg)	**Dose** (route)	**Time** (hr)	**Plasma** Mean (ng/mL)	SD	**Testis** Mean (ng/mL)	SD
50	I.P.	0	BQL	NA	BQL	NA
	BID 12 hr interval	0.5	11648	759	26755	2665
		1	7984	1630	26212	6675
		2	5393	1685	11153	1777
		4	1696	482	2749	730
		8	198	108	529	213
		12	25.0	13.5	118	87.4
		12.5	9949	723	20833	7324
		13	5211	265	14262	4127
		14	2990	1000	7385	2083
		16	565	312	1275	657
		20	8.55	7.06	54.1	63.6
		24	1.28	0.348	6.49	NA

**PK parameters**	**Unit**	**Plasma**	**Testis**
T_{max}	hr	0.5	0.5
C_{max}	ng/mL	11650	26760
Terminal $t_{1/2}$	hr	0.865	0.981
AUC_{0-12hr}	hr*ng/mL	25830	60360
$AUC_{12-24hr}$	hr*ng/mL	15106	36270
AUC_{0-24hr}	hr*ng/mL	40937	96640
AUC_{INF}	hr*ng/mL	40938	96650
$AUC_{testical}/AUC_{plasma}$	%	N/A	236

Abbrevations: BQL (below the quantifiable limit of 1.00 ng/mL), NA (not available), SD (standard deviation).

Table S5. Downregulation of Multiple Germ Cell Expressed Genes upon Treatment with JQ1, Related to Figure 5

	Ccna1	*Msy2*	*Plk1*	*Aurkc*	*Akap4*
6 wks JQ1, QD	-3.0x	-2.5x	-5.3x	-2.7x	-2.3x
3 wks JQ1, BID	-2.2x	-4.4x	-2.4x	-4.0x	-5.2x

Table S6. Primers for Quantitative PCR, Related to Figure 5

Gene name	Forward Primer	Reverse Primer	Reference for mRNA expression
Akap4	GTCAGAAGGCGAGTTAAATCTGG	ATCCCTCCGTCTTAGACTGGT	Miki et al., 2002
Aurkc	GGGCGTGTGTACTTGGCTC	AAGTTGGTGCTCCAATCCCTC	Tang et al., 2006
Brdt	GCTTTGGGACTCCACAACTACTATG	GATTGTCCATTTTCCCCTTGATC	Shang et al., 2004
Ccna1	TTTCCCCAATGCTGGTTGA	AACCAAAATCCGTTGCTTCCT	Sweeney et al., 1996
Hist1h1t	GCTGATTCCTGAGGCCCTTT	CAGGGCAGCAAGGGACAT	Shang et al., 2007
Klf17	CCTCCCGTTTGTTCTCAACTTG	GGTGCATAGCCTGTTCCTTATTG	Yan et al., 2002
Msy2	CATCCTTATTGTTCCGAGGCA	GGAGGTATGAGCTGGCTGGTT	Gu et al., 1998
Papolb	CGCCAACAGAGAAACAACATTTAG	CCAACCAGGATTCGGATCTTT	Kashiwabara et al., 2000
Plk1	CGAGGATCTGGAGGTGAAAA	AGGAGTGCCACACAAGGTCT	Matsubara et al., 1995
Plzf	TGGAGAAGCATTTGGGTATCTACTC	AAGACGGCATGCTCAACACA	Buaas et al., 2004; Costoya et al., 2004
Prm1	TGCACAGAATAGCAAGTCCATCA	TGTGGCGAGATGCTCTTGAA	Kleene et al., 1984
Stra8	GAGTGAGGCCCAGCATATGTC	CCTCTGGATTTTCTGAGTTGCA	Zhou et al., 2008

Table S7. Pharmacokinetic Study of JQ1 (50 mg/kg IP) in Rats, Related to Figure 5

**Dose** (mg/kg)	**Dose** route	**Time** (hr)	**Plasma** Mean (ng/mL)	SD
50	IP	0	BQL	NA
		0.5	6953	1701
		1	6294	1229
		2	3979	513
		4	2217	360
		6	911	322
		8	317	124
		12	25.0	10.9
		24	BQL	NA

**PK parameters**	**Unit**	**Mean**	**SD**
T_{max}	hr	0.500	0.000
C_{max}	ng/mL	6953	1701
Terminal $t_{1/2}$	hr	1.15	0.0307
AUC_{last}	hr*ng/mL	21422	3599.4
AUC_{INF}	hr*ng/mL	21463	3617.7

Data S1. MAFFT Alignment of Human BRDT and Human BRD4, Related to Figure 1

```
BRDT            M-------------------------------SLPSRQTAIIVNPPPPEYINTKKNGRLT
BRD4            MSAESGPGTRLRNLPVMGDGLETSQMSTTQAQAQPQPANAASTNPPPPETSNPNKPKRQT
                *                               : *.  .*  .******  *.:*  * *

BRDT            NQLQYLQKVVLKDLWKHSFSWPFQRPVDAVKLQLPDYYTIIKNPMDLNTIKKRLENKYYA
BRD4            NQLQYLLRVVLKTLWKHQFAWPFQQPVDAVKLNLPDYYKIIKTPMDMGTIKKRLENNYYW
                ****** :**** ****.*:****:*******:*****.***.***:.********:**

BRDT            KASECIEDFNTMFSNCYLYNKPGDDIVLMAQALEKLFMQKLSQMPQEEQVVGVKERIKKG
BRD4            NAQECIQDFNTMFTNCYIYNKPGDDIVLMAEALEKLFLQKINELPTEETEIMIVQAKGRG
                :*.***:******:***:************:******:**:.::* **  : : :   :*

BRDT            ------------------------------TQQNIAVSSAKEKSSPSATEKVFKQQEIPS
BRD4            RGRKETGTAKPGVSTVPNTTQASTPPQTQTPQPNPPPVQATPHPFPAVTPDLIVQTPVMT
                                              .* * .  .*. :. *:.* .:: *  : :

BRDT            VFPKTSI----------------SPLNVVQGASVNSSSQTAAQVTKGVKRKADTTTPAT-
BRD4            VVPPQPLQTPPPVPPQPQPPPAPAPQPVQSHPPIIAATPQPVKTKKGVKRKADTTTPTTI
                *.*  .:                :*  * . ..: :::  ..:..************:*

BRDT            SAVKASSEFSP-----TFTEKSVALPPIKENMPKNVLPDSQQQYNVVKTVKVTEQLRHCS
BRD4            DPIHEPPSLPPEPKTTKLGQRRESSRPVKP--PKKDVPDSQQHPAPEKSSKVSEQLKCCS
                ..:: ...:.*     .: ::  :  *:*   **: :*****:    *: **:***: **

BRDT            EILKEMLAKKHFSYAWPFYNPVDVNALGLHNYYDVVKNPMDLGTIKEKMDNQEYKDAYKF
BRD4            GILKEMFAKKHAAYAWPFYKPVDVEALGLHDYCDIIKHPMDMSTIKSKLEAREYRDAQEF
                 *****:**** :******:****:*****:* *::*:***:.***.*:: :**:** :*
```

```
BRDT          AADVRLMFMNCYKYNPPDHEVVTMARMLQDVFETHFSKIPIEPVE-----SMPLCYIKTD

BRD4          GADVRLMFSNCYKYNPPDHEVVAMARKLQDVFEMRFAKMPDEPEEPVVAVSSPAVPPPTK

              .******* *************:*** ****** :*:*:* ** *     * *     *.


BRDT          ITE--TTGRENTNEASSEGNSSDDSEDERVKRLAKLQEQLKAVHQQLQVLSQVPFRKLNK

BRD4          VVAPPSSSDSSSDSSSDSDSSTDDSEEERAQRLAELQEQLKAVHEQLAALSQ-PQQNKPK

              :.   ::. ..::.:*....*:****:**.:***:*********:** .*** * ::  *


BRDT          KKEKSKKEKKKEKVNNSNENPRKMCEQMRLKEKSKRNQPKKRKQQFIG------------

BRD4          KKEKDKKEKKKEK------HKRKEEVEENKKSKAKEPPPKKTKKNNSSNSNVSKKEPAPM

              ****.********      : **   : . *.*:*.  *** *::  .


BRDT          -------LKSEDEDNAKPMNYDEKRQLSLNINKLPGDKLGRVVHIIQSREPSLSNSNPDE

BRD4          KSKPPPTYESEEEDKCKPMSYEEKRQLSLDINKLPGEKLGRVVHIIQSREPSLKNSNPDE

                      :**:**:.***.*:*******:******:****************.******


BRDT          IEIDFETLKASTLRELEKYVSACLRKRPLKPPAKKIMMSKEELHSQKKQELEKRLLDVNN

BRD4          IEIDFETLKPSTLRELERYVTSCLRKK-RKPQAEKV--------------------DV--

              *********.*******:**::****:  ** *:*:                    **


BRDT          QLNSRKRQTKSDKTQPSKAVENVSRLSESSSSSSSSSESESSSSDLSSSDSSDSESEMFP

BRD4          ------------------------IAGSSKMKGFSSSESESSSES-SSSDSEDSETEMAP

                                        ..*. .. *********.. *****.***:** *


BRDT          K-----------------------------------------------------------

BRD4          KSKKKGHPGREQKKHHHHHHQQMQQAPAPVPQQPPPPPQQPPPPPPPPQQQQQPPPPPPP

              *
```

```
BRDT            ---------------------------------------FTE------------------
BRD4            SMPQQAAPAMKSSPPPFIATQVPVLEPQLPGSVFDPIGHFTQPILHLPQPELPPHLPQPP
                                                       **:

BRDT            ------------------------------------------------------------
BRD4            EHSTPPHLNQHAVVSPPALHNALPQQPSRPSNRAAALPPKPARPPAVSPALTQTPLLPQP

BRDT            ----------------------------------VKP-----------------------
BRD4            PMAQPPQVLLEDEEPPAPPLTSMQMQLYLQQLQKVQPPTPLLPSVKVQSQPPPPLPPPPH
                                                  *:*

BRDT            ------------------------------------------------------------
BRD4            PSVQQQLQQQPPPPPPPQPQPPPQQQHQPPPRPVHLQPMQFSTHIQQPPPPQGQQPPHPP

BRDT            ---------------------------------------------------------NDS
BRD4            PGQQPPPPQPAKPQQVIQHHHSPRHHKSDPYSTGHLREAPSPLMIHSPQMSQFQSLTHQS
                                                                         ::*

BRDT            PSKENVKKMKNECIPPEGRTGVTQIGYCVQDTTSANTTLVHQTTPSHVMPPNHHQLAFNY
BRD4            PPQQNVQPKKQEL---RAASVVQPQPLVVVKEEKIHSPIIRSEPFSPSLRPEPPKHPESI
                *.::**:  *:*    .. : *      * .  . ::.:::. . *  : *:  : . .

BRDT            QELEHLQTVKNISPLQ-----ILPPSGDSEQLSNGITVMHPSGDSDTTMLESECQAPV--
BRD4            KAPVHLPQRPEMKPVDVGRPVIRPPEQNAP----------PPGAPDKDKQKQEPKTPVAP
                :   **    ::.*::     * **. ::           *.* .*.   :.* ::**

BRDT            QKDIKIKNADSWKSL--GKPVKPSGVMKSSDELFNQFRKAAIEKEVKARTQELIRKHLEQ
```

```
BRD4            KKDLKIKNMGSWASLVQKHPTTPSSTAKSSSDSFEQFRRAAREKEEREKALKAQAEHAEK
                :**:**** .** **   :*..**.. ***.: *:***:** *** : :: :   :* *:

BRDT            NTKELKASQENQRDLGNGLTV------ESFSNKIQNKCSGEEQKEHQQSSEA--------
BRD4            EKERLRQERMRSREDEDALEQARRAHEEARRRQEQQQQQRQEQQQQQQQQAAAVAAAATP
                :.:.*: .: ..*:  :.*        *:  .: *:: . :**:::**.. *

BRDT            ---QDKSKLWLLKDRDLARQKEQERRRRREAMVGTIDMTLQSDIMTMFENNFD
BRD4            QAQSSQPQSMLDQQRELARKREQERRRRREAMAATIDMNFQSDLLSIFEENLF
                   ..:.:  * ::*:***::**********..****.:***::::**:*:
```

Data S2. MAFFT Alignment of Human BRDT and Mouse BRDT. Related to Figure 1

```
hBRDT           MSLPSRQTAIIVNPPPPEYINTKKNGRLTNQLQYLQKVVLKDLWKHSFSWPFQRPVDAVK

mBRDT           MSLPSRQTA-IVNPPPPEYINTKKSGRLTNQLQFLQRVVLKALWKHGFSWPFQQPVDAVK

                ********* **************.********:**:**** ****.******:******


hBRDT           LQLPDYYTIIKNPMDLNTIKKRLENKYYAKASECIEDFNTMFSNCYLYNKPGDDIVLMAQ

mBRDT           LKLPDYYTIIKTPMDLNTIKKRLENKYYEKASECIEDFNTMFSNCYLYNKTGDDIVVMAQ

                *:*********.**************** *********************.*****:***


hBRDT           ALEKLFMQKLSQMPQEEQVVGVKERIKKGTQQNIAVSSAKEKSSPSATEKVFKQQEIPSV

mBRDT           ALEKLFMQKLSQMPQEEQVVGGKERIKKDIQQKIAVSSAKEQIPSKAAENVFKRQEIPSG

                ********************* ******. **:********: ...*:*:***:*****


hBRDT           FPKTSISPLNVVQGASVNSSSQTAAQVTKGVKRKADTTTPATSAVKASSEFSPTFTEKSV

mBRDT           LPDISLSPLNMAQEAPPICDSQSLVQITKGVKRRADTTTPTTSIAKASSESPPTLRETKP

                :*. *:****:.* *.  ..**: .*:******:******:** .***** .**: *..


hBRDT           ALPPIKENMPKNVLPDSQQQYNVVKTVKVTEQLRHCSEILKEMLAKKHFSYAWPFYNPVD

mBRDT           VNMPVKENTVKNVLPDSQQQHKVLKTVKVTEQLKHCSEILKEMLAKKHLPYAWPFYNPVD

                .  *:***  **********::*:*********:**************:.**********


hBRDT           VNALGLHNYYDVVKNPMDLGTIKEKMDNQEYKDAYKFAADVRLMFMNCYKYNPPDHEVVT

mBRDT           ADALGLHNYYDVVKNPMDLGTIKGKMDNQEYKDAYEFAADVRLMFMNCYKYNPPDHEVVA

                .:********************* ***********:***********************:


hBRDT           MARMLQDVFETHFSKIPIEPVESMPLCYIKTDITETTGRENTNEASSEGNSSDDSEDERV

mBRDT           MARTLQDVFELHFAKIPDEPIESMHACHLTTNSAQALSRESSSEASSGDASSEDSEDERV

                *** ****** **:*** **:***  *::.*: ::: .**.:.**** . **:*******
```

```
hBRDT          KRLAKLQEQLKAVHQQLQVLSQVPFRKLNKKKEKSKKEKKKEKVNNSNENPRKMCEQMRL
mBRDT          QHLAKLQEQLNAVHQQLQVLSQVPLRKLKKKNEKSKRAPKRKKVNNRDENPRKKPKQMKQ
               ::********:*************:***:**:****:  *::**** :*****  :**:

hBRDT          KEKSKRNQPKKRKQQFIGLKSEDEDNAKPMNYDEKRQLSLNINKLPGDKLGRVVHIIQSR
mBRDT          KEKAKINQPKKKKPL---LKSEEEDNAKPMNYDEKRQLSLDINKLPGDKLGRIVHIIQSR
               ***:* *****:*     ****:*****************:***********:*******

hBRDT          EPSLSNSNPDEIEIDFETLKASTLRELEKYVSACLRKRPLKPPAKKIMMSKEELHSQKKQ
mBRDT          EPSLRNSNPDEIEIDFETLKASTLRELEKYVLACLRKRSLKPQAKKVVRSKEELHSEKKL
               **** ************************** ******.*** ***:: *******:**

hBRDT          ELEKRLLDVNNQLNSRKRQTK------------------------------SDKTQPSKA
mBRDT          ELERRLLDVNNQLNCRKRQTKRPAKVEKPPPPPPPPPPPPPPPELASGSRLTDSSSSSGS
               ***:**********.******                              :*.:..* :

hBRDT          VENVSRLSESSSSSSSSSESESSSSDLSSSDSSDSESEMFPKFTEVKPNDSPSKENVKKM
mBRDT          GSGSSSSSSGSSSSSSSSGSASSSSDSSSSDSSDFEPEIFPKFTGVKQNDLPPKENIK--
                 .. *  *..******** * ***** ******* *.*:***** ** ** *.***:*

hBRDT          KNECIPPEGRTGVTQIGYCVQDTTSANTTLVHQTTPSHVMPPNHHQLAFNYQELEHLQTV
mBRDT          --------------QIQSSVQDITSAEAPLAQQSTAPCGAPGKHSQQMLGCQVTQHLQAT
                             **  .*** ***::.*.:*:*..   * :* *  :. *  :***:.

hBRDT          KNISPLQILPPSGDSEQLSNGITVMHPSGDSDTTMLESECQAPVQKDIKIKNADSWKSLG
mBRDT          ENTASVQTQPLSGDCKRVLLGPPVVHTSAES-LTVLEPECHAPAQKDIKIKNADSWKSLG
               :* :.:*  * ***.:::  * .*:*.*.:*  *:**.**:**.***************
```

```
hBRDT          KPVKPSGVMKSSDELFNQFRKAAIEKEVKARTQELIRKHLEQNTKELKASQENQRDLGNG
mBRDT          KPVKASSVLKSSDELFNQFRKAAIEKEVKARTQEQMRKHLEHNAKDPKVSQENQREPGSG
               ****.*.*:************************* :*****:*:*: *.******: *.*

hBRDT          LTVESFSNKIQNKCSGEEQKEHQQSSEAQDKSKLWLLKDRDLARQKEQERRRREAMVGTI
mBRDT          LTLESLSSKVQDKSLEEDQSEQQPPSEAQDVSKLWLLKDRNLAREKEQERRRREAMAGTI
               **:**:*.*:*:*.  *:*.*:* .***** *********:***:***********.***

hBRDT          DMTLQSDIMTMFENNFD
mBRDT          DMTLQSDIMTMFENNFD
               *****************
```
